# Photocontrolled Release of Urea Enables the Detection of Urea–Urease Intermediates by Cryo‐FTIR

**DOI:** 10.1002/anie.202504332

**Published:** 2025-07-25

**Authors:** Caterina G.C. Marques Netto, Sarah Bell, Caio B. Castro, Pedro Henrique Machado, Vatsal Purohit, Katherine M. Davis, Ana Paula L. Batista, R. Brian Dyer

**Affiliations:** ^1^ Departamento de Quimica Universidade Federal de São Carlos Rod. Washington Luiz, Km 235, s/n São Carlos SP 13565–905 Brazil; ^2^ Department of Chemistry Emory University 1515 Dickey Drive Atlanta GA 30030 USA

**Keywords:** Caged urea, Cryo‐FTIR, Photocontrolled delivery, Reaction intermediated, Urease

## Abstract

Caged compounds are a valuable tool to photocontrol the release of enzymatic substrates in a reaction. For example, caged urea molecules were proposed to control urea delivery to urease reactions and aid the determination of the catalytic mechanism of this enzyme. Owing to the success of several caged compounds based on ruthenium complexes, we synthesized a new inorganic caged urea (RuBpy‐urea) and monitored the release of free urea by infrared spectroscopy upon irradiation. Transient infrared absorption (TRIR) indicates diffusion‐controlled release of urea, and kinetics studies after photolysis demonstrate the functionality of the new caged molecule for photocontrolled delivery of urea to urease. Coupling photocontrolled delivery of urea to cryo‐FTIR (fourier transform infrared spectroscopy) revealed that inhibition of urease results in different FTIR spectrum than the one observed for the noninhibited urease.

## Graphical abstract

Urease is a fast enzyme, presenting *k*_cat_ of the order of 10^3^–10^4^ s^−1^. To enable the visualization of its mechanism we explored the technique of cryo‐FTIR. By means of using a ruthenium complex as a caged‐urea, upon light irradiation urea was released and we could see the coordination of the urea to the active site.

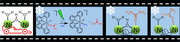

## Introduction

Light absorption is a useful method to trigger chemical reactions, providing the energy necessary to overcome activation barriers on a short timescale to initiate the reaction concertedly. Some biomolecular reactions naturally lend themselves to this approach because they involve light harvesting and energy transduction. ^[^ ^1^, ^2^, ^3^, ^4^, ^5^ ^]^ The photophysical and biochemical properties of these biomolecules are easily tuned enabling the systematic study of these systems using light to initiate and probe the dynamics of the processes of interest. ^[^ ^6^, ^7^, ^8^, ^9^ ^]^ However, biomolecular reactions that are not natively light‐dependent do not have this flexibility and are harder to study. For instance, many enzymatic processes occur on the submillisecond time frame that cannot be readily studied by conventional stopped flow approaches and intermediates have been proposed with a high level of uncertainty.^[^ ^10^, ^11^, ^12^ ^]^ To overcome this limitation, photolabile “caged compounds” have been developed, with the ability to liberate a trapped reactant molecule upon light irradiation.^[^ ^13^ ^]^ Light‐driven release of a caged substrate enables exquisite control of a biochemical process, ^[^ ^14^ ^]^ as the activation of the reaction can be performed at a precise time and faster than the reaction dynamics of interest.^[^ ^15^ ^]^ Thus, any enzyme reaction has the possibility of being studied by *light‐minus‐dark* difference spectroscopy providing the substrate is released by a rapid and uniform photolysis of an inactive photolabile analogue.^[^ ^16^, ^17^, ^18^, ^19^ ^]^ For example, cryophotolysis of ((nitrophenyl)ethyl)‐caged ATP (adenosine triphosphate) coupled with X‐ray crystallography and microspectrophotometry was employed to study the ATP‐dependent enzymatic reaction of thymidylate kinase from *Mycobacterium tuberculosis*.^[^ ^20^ ^]^ In another study, time‐resolved femtosecond crystallography was used in conjunction with photosensitive caged‐compounds to track an NO‐bound form of a fungal NO reductase.^[^ ^21^ ^]^ Thus, the utility of photolabile caged reagents for the characterization of catalytic intermediates is clear, but none are available for many enzymes.

**Figure 1:**
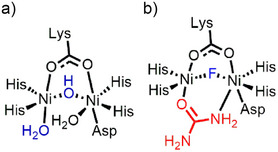
a) Urease active site and b) the intermediate observed in the fluoride inhibited urease.

A biological catalyst that could benefit from this approach is urease, as its catalytic mechanism has been long debated. ^[^ ^22^ ^]^ Urease is responsible for the hydrolysis of urea to CO_2_ and ammonia, and has medical relevance owing to its role as a general microbial virulence factor.^[^ ^23^ ^]^ The active site of the enzyme has two nickel ions, each coordinated to two histidine residues, a carbamylated lysine, a water molecule, and a bridged hydroxide.^[^ ^24^, ^25^ ^]^ One of these nickel centers, Ni(2), is also coordinated to an aspartate, generating a penta‐coordinated Ni(1) and a hexa‐coordinated Ni(2), ^[^ ^24^, ^25^, ^26^, ^27^ ^]^ as shown in Figure 1a. The seminal work of Mazzei et al. identified a bridged O,N coordinated urea intermediate in a fluoride‐inhibited urease X‐ray crystal structure (Figure 1b),^[^ ^12^ ^]^ however, the mechanism of urease and urea coordination to the uninhibited enzyme is still unknown. The short lifetime of the urease–urea Michaelis complex hinders visualization of the proposed catalytic intermediates.^[^ ^12^ ^]^ Furthermore, the use of caged urea has been proposed as a possible methodology to detect intermediates in the urease mechanism.^[^ ^28^ ^]^ It should be possible to detect urease intermediates by FTIR spectroscopy, since the urea carbonyl stretching band is sensitive to the environment and to coordination.^[^ ^29^, ^30^, ^31^, ^32^, ^33^, ^34^, ^35^, ^36^, ^37^, ^38^, ^39^, ^40^ ^]^ Thus, FTIR spectroscopy of urea coordination to the active site of urease can serve as a useful probe of catalytic turnover.

Previously, photolabile caged urea compounds based on α‐substituted 2‐nitrobenzyl groups covalently attached to the urea nitrogen were developed. ^[^ ^41^ ^]^ Surprisingly, however, there are no reports in the literature of these compounds being used to study the mechanism of the urease enzyme. Possible shortcomings of these compounds that may have precluded such an application include inhibition of urease by either the caged urea or its photolytic side products, poor uncaging quantum yields, or slow uncaging of urea with respect to urease reaction steps.

Motivated by the need for a more effective caged urea reagent to enable determination of the urease mechanism, and taking inspiration from the development of a related caged reagent, RuBi‐GABA (Gamma‐aminobutyric acid),^[^ ^42^ ^]^ we synthesized a ruthenium based caged‐urea. Cryogenic techniques are significant tools to trap and characterize the interaction of adducts of biorelevant molecules,^[^ ^43^, ^44^ ^]^ thus, we coupled photouncaging with cryo‐FTIR to trap urea–urease reaction intermediates. Photoreleased urea was shown to react with the enzyme, indicating that this new caged urea can be used to initiate the reaction with light and thereby facilitate cryo‐trapping and time‐resolved studies of the uninhibited urease mechanism.

## Results and Discussion

### RuBpy‐Urea Synthesis and Characterization

**Scheme 1:**
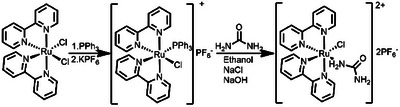
Synthesis of RuBpy–urea. cis‐Ru(bpy)_2_Cl_2_ reacts with triphenylphosphine to generate the [Ru(bpy)_2_PPh_3_Cl]PF_6_ intermediate, which is isolated as [Ru(bpy)_2_PPh_3_Cl]PF_6_. The reaction of this intermediate with urea in an ethanolic solution containing NaCl and NaOH results in the formation of RuBpy–urea with 5% yield.

The ruthenium‐based caged urea was inspired by RuBi‐GABA, ^[^ ^42^ ^]^ developed to photodeliver the neurotransmitter GABA by a metal–ligand heterolytic cleavage. ^[^ ^45^, ^46^, ^47^ ^]^ Several amines have already been caged using the same strategy. ^[^ ^48^ ^]^ Therefore, *cis‐*Ru(bpy)_2_Cl_2_ was first reacted with triphenylphosphine to obtain *cis*‐[Ru(bpy)_2_PPh_3_Cl]PF_6_,^[^ ^46^ ^]^ which was then further reacted with urea to produce [Ru(bpy)_2_ClUrea]PF_6_ (RuBpy‐Urea) in 5% yield as shown in Scheme 1.

Formation of RuBpy–urea was confirmed by ^1^H, ^13^C, ^31^P nuclear magnetic resonance (NMR), high resolution mass spectrometry (HRMS), microanalysis, and UV‐vis spectroscopy (Figures S1–S6). Although RuBi‐GABA has triphenylphosphine (PPh_3_) from the precursor in its structure, ^[^ ^42^ ^]^ it is not present in RuBpy–urea, as evidenced by the HRMS, ^1^H NMR, and ^31^P NMR. The absence of PPh_3_ indicates that urea coordination depends on the labilization of PPh_3_ to bind to its position. This might reduce the quantum yield, since phosphine ligands were shown to produce a longer emission lifetime and increase the quantum yield. ^[^ ^49^, ^50^ ^]^ Electronic spectroscopy of RuBpy–urea (Figure S7), reveals a metal‐to‐ligand charge transfer (MLCT) band ^[^ ^51^ ^]^ centered at 488 nm (*ε*_488 _= 6700 Lmol^−1^cm^−1^, *ε*_527 _= 4012 Lmol^−1^cm^−1^). The value of ε_527_ was employed in the determination of the quantum yield of photolysis.

Synthesis of the isotope‐labeled caged compound with ^15^N‐urea and ^13^C‐urea enabled the assignment of the signals specific to the coordinated urea in the ^15 ^N (Figure S4) and ^13^C NMR (Figure S5). From these spectra, it is evident that urea is coordinated through the nitrogen atom, owing to the presence of two sets of triplets in the ^15 ^N NMR. However, the ^13^C NMR of the ^13^C‐labeled RuBpy‐urea revealed the presence of 4 signals between 165–155 ppm, indicating that it is possible that a mixture of carbonyl and nitrogen coordinated (protonated and deprotonated) urea is present. Another possibility is *cis‐trans* photoisomerization due to light exposure during sample preparation,^[^ ^52^ ^]^ which has been observed for several ruthenium complexes. ^[^ ^53^, ^54^ ^]^ To gain further insight into this photoisomerization process and to assist with IR band assignments, density functional theory (DFT) calculations were carried out. The difference in energy between the *cis‐* and *trans‐*isomers of RuBpy–urea is 13.4 kcal mol^−1^ (Figure S8), which might be reached by photoexcitation in a photoisomerization reaction.^[^ ^54^ ^]^ Thus, it is possible that the caged urea might be a mixture of these isomers.

The Ru–urea bond was shown to be labile, as the signal of free urea was observed in the ^13^C NMR (159 ppm), revealing the ease of urea substitution by other molecules. Therefore, the choice of solvent for the photolysis experiments must be careful to avoid release of free urea by ligand substitution. Considering this property of the caged urea and its poor water solubility, a cosolvent is required that is compatible with enzyme stability and function but will not displace urea in the dark. Urease is unstable in ethanol, glycerol, DMF (dimethylformamide), pyridine^[^ ^55^ ^]^ and DMSO (dimethyl sulfoxide). ^[^ ^55^, ^56^ ^]^ As urease was extracted and crystallized from 32% acetone in water in the work of Sumner, ^[^ ^57^ ^]^ and the enzyme was shown to maintain initial reaction rates at concentrations up to 85% acetone in water, ^[^ ^55^ ^]^ we decided to employ acetone as a cosolvent in the photolysis experiments. Moreover, RuBpy–urea is stable up to 2 h in acetone/buffer mixture (Figure S9).

### RuBpy–Urea Photolysis ‐FTIR Detection

**Figure 2:**
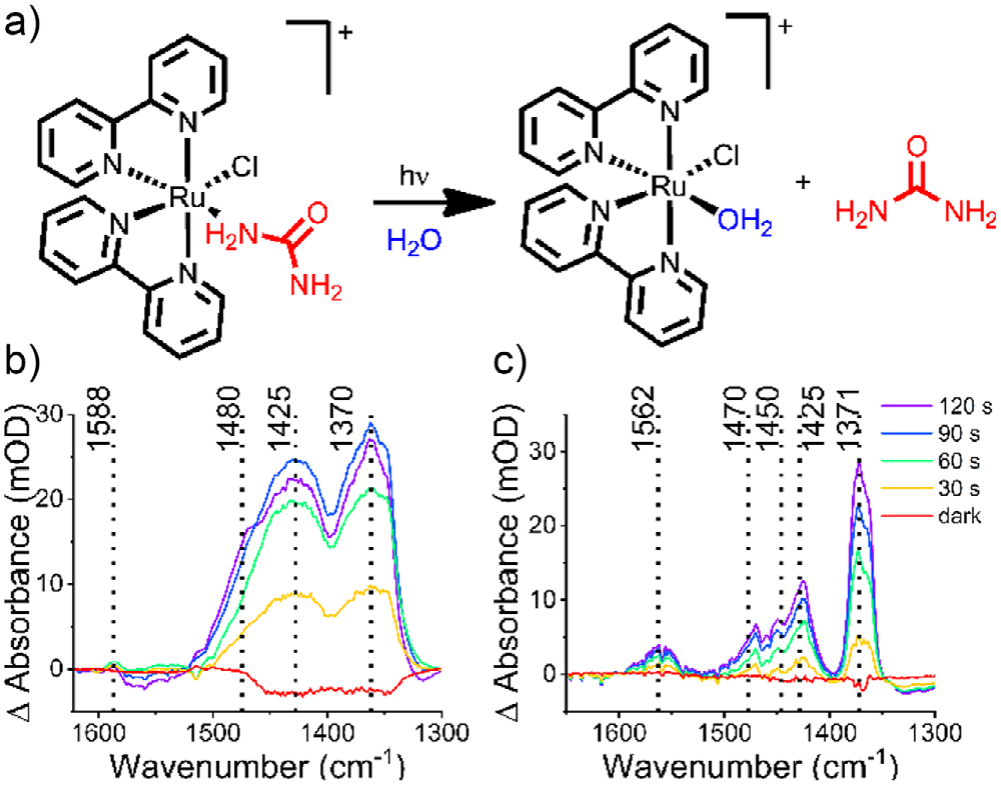
Phototitration of RuBpy–urea a). Caged‐urea photolysis monitored by FTIR spectroscopy of ^12^C‐RuBpy‐urea b) and ^13^C‐RuBpy‐urea c). All spectra were ratioed against a dark spectrum. The experiments are obtained from photolysis of a thin film of the RuBpy–urea solution (in 75:25 (v/v) Tris‐DCl (50 mM pD 7.3) and acetone) in a FTIR cell composed of CaCl_2_ windows and 50 µm Teflon spacer. Photolysis was performed with a Nd:YLF laser *λ* = 527 nm (40.5 mW).

As stated previously, polypyridine ruthenium complexes exhibit a *cis‐trans* interconversion due to photo and/or thermal excitations. ^[^ ^52^ ^]^ For instance, excitation of the *cis* complex at its MLCT transitions enables the photodetachment of monodentate ligands in Ru–polypyridine complexes followed by photoisomerization to yield the *trans* isomer.^[^ ^54^ ^]^ If another molecule replaces the detached ligand, a new complex will form. Similarly, to other ruthenium polypyridine caged complexes, photolysis of RuBpy–urea can be performed by irradiating at the MLCT band^[^ ^58^ ^]^ to yield formation of the aquo complex and the release of free urea (Figure 2).

^1^H NMR experiments before and after light exposure revealed that a broad signal at 8.27 ppm, ascribed to urea NH_2_ in the dark spectrum, disappears upon light exposure (Figure S10), indicating that urea is released from RuBpy–urea after irradiation with light. This uncaging of urea can also be monitored by FTIR spectroscopy using the characteristic νCO and νCN bands of urea centered around 1605 and 1490 cm^−1^ (Figure S11). For instance, we observe the emerge of bands at 1588 and 1480 cm^−1^ for ^12^C RuBpy–urea (Figure 2b).^[^ ^59^ ^]^ The ≈10 cm^−1^ difference between experiments is most probably due to the presence of RuBpy in the uncaging experiments, which can alter the solvent polarity and consequently, the band position in the FTIR spectrum. When ^13^C‐labeled RuBpy–urea is photolyzed, bands at 1562 cm^−1^ (νCO) and 1450 cm^−1^ (νCN) are observed (Figure 2c). This difference is the isotope shift expected for ^13^C labeling of urea (Figure S11), evidence that these IR peaks are due to uncaging of urea from RuBpy–urea upon irradiation. As observed in Figure 2, the νCO band at 1588 cm^−1^ observed in the photolysis of the unlabeled caged urea has a lower intensity than the νCO of the ^13^C labeled one. This can be explained due to the partial overlap of the solvent band (water and acetone) in this region, whereas the ^13^C free urea has a band at a clearer region, enabling its visualization. The region between 1630 and 1520 cm^−1^ for the ^12^C‐caged urea photolysis can be better observed in the Supporting Information (Figure S12).

In the phototitration experiments, the emergence of bands related to formation of the [Ru(bpy)_2_Cl(D_2_O)]^+^ complex should also be observed. Bands at 1425 and 1370 cm^−1^ (Figure 2b) emerge upon photolysis of ^12^C‐RuBpy–urea. This region is associated with the νC═C and νC═N of the bpy ligand.^[^ ^60^, ^61^ ^]^ There is also a strong overlapping IR band due to ν_as_(CN) of free urea at 1486 cm^−1^. Closely related bands emerge in the photolysis of ^13^C‐RuBpy–urea (1470, 1425, and 1371 cm^−1^), as seen in Figure 2c, indicating that these bands are from the formation of the [Ru(bpy)_2_Cl(D_2_O)]^+^ complex. In this case, the ν_as_(CN) of free ^13^C‐urea shifts down to 1450 cm^−1^, where it is more distinct from the bpy bands.

To confirm these assignments, vibrational analysis of *cis*‐[Ru(bpy)_2_Cl(D_2_O)]^+^ via DFT calculations (Figure S13) reveals vibrational modes localized at the bpy ligand around 1500 cm^−1^. Similar vibrational modes are also observed for the RuBpy–urea complex. As the D_2_O bending mode is around 1170 cm^−1^ and the related coordinated stretching modes were found to be around 2700 cm^−1^ region in DFT calculations, they are not shown in Figure S13. The molar absorptivity of the bands is higher for cis‐[Ru(bpy)_2_Cl(D_2_O)]^+^ than for RuBpy‐urea, therefore, the differential spectra between these species would lead to the emergence of bpy vibrations, confirming that upon photolysis, free urea and [Ru(bpy)_2_Cl(D_2_O)]^+^ are formed.

Since it might be possible to trap intermediate states in the urease catalytic cycle at low temperature, the photolysis of RuBpy–urea was also investigated at 70 K (Figure S14). Uncaged urea was observed at 70 K by the emergence of the peak at 1604 cm^−1^. By monitoring the absorption of this peak as a function of illumination time, and considering the molar absorptivity of the urea carbonyl as 590 M^−1^ cm^−1^,^[^ ^62^ ^]^ it was possible to calculate the quantum yield (QY) of photolysis (Equation 1, where *E*_photon_ is the energy of a photon at the excitation wavelength of 527 nm and *N*_A_ is Avogadro's number). We obtained a value of 0.04% at 70 K and 0.13% at room temperature. These values agree with the QYs obtained for different caged forms of GABA.^[^ ^46^ ^]^

(1) $$\begin{array}{c} QY\;=\;\frac{moles\;of\;radical}{moles\;of\;absorbed\;photons}*100=\frac{moles\;of\;urea}{\left(\frac{(Power\;\left(W\right)*\mathrm{time}\left(s\right)}{E_{photon}\left(J\right)}\right)*fraction\;of\;photons\;absorbed\;vs\;total*\left(1-scatter\right)*\left(\frac{1}{N_{A}}\right)\;} \end{array}$$

### Transient Infrared Absorption

**Figure 3:**
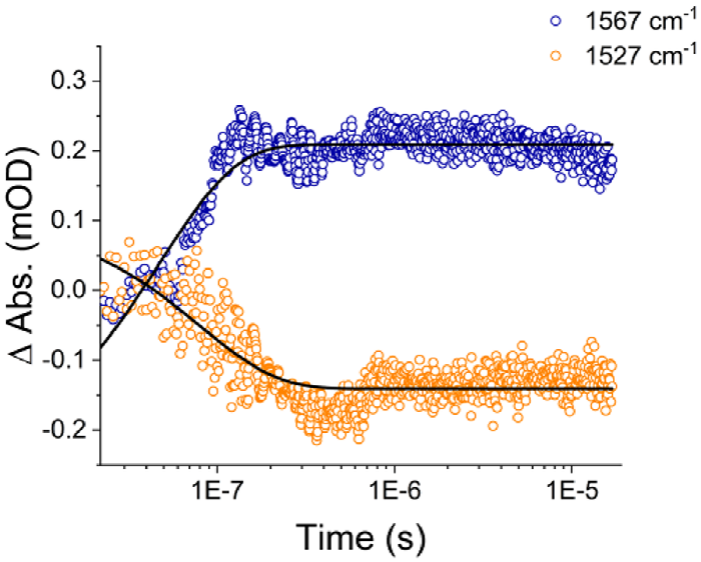
Room temperature TRIR photolysis transients of ^13^C RuBpy‐Urea. Laser photolysis of caged urea was performed using a 532 nm laser pulse (10 ns) and monitoring the IR transient absorbance at 1567(free urea, blue circles) and at 1527 cm‐1 (caged urea, orange circles); black traces are exponential fits with *τ* of 80 ns. A single laser pulse (Spectra Physics GCR‐3 Nd:YAG, 532 nm; ca 100–500 µJ; 10 ns duration) initiated the reaction.

Uncaging of caged compounds is preceded by the formation of an excited state M* upon light excitation, occurring on a timescale of <1 ps. The photoproducts, however, are generated in dark reactions, which are comparatively slow. Kinetics vary widely from nanoseconds to hundreds of milliseconds.^[^ ^63^ ^]^ It is necessary to know the kinetics of urea release to evaluate if RuBpy–urea can be used for urease assays. Ideally, the uncaging reaction should be complete at least an order of magnitude faster than the timescale for formation of the first catalytic intermediate (the Michaelis complex), such that the full catalytic cycle can be observed kinetically.^[^ ^13^ ^]^ Therefore, we performed flash photolysis kinetics of ^13^C RuBpy–urea and monitored urea release by TRIR spectroscopy (Figure 3). These experiments reveal a bleach at 1527 cm^−1^ and the development of an absorbance feature at 1567 cm^−1^. These frequencies correspond to the νCO bands of caged and free urea, respectively. The data were fit to a single exponential function with a lifetime τ for both the bleach and transient absorbance of 80 ns. This timeframe indicates that photorelease of urea is a diffusion‐controlled process. For comparison, other caged‐ureas were reported to release urea on timescales of 10–100 µs^[^ ^28^, ^41^ ^]^ Considering that the slowest step of an enzymatic reaction governs *k*_cat_,^[^ ^64^ ^]^ and that wild‐type ureases have *k*_cat_ values between 10^3^ and 10^4^ s^−1^,^[^ ^26^, ^65^ ^]^ it is necessary that the uncaging of urea happens faster than 100 µs to capture the rate determining step, and perhaps even faster to capture early intermediates. Therefore, uncaging of urea in RuBpy–urea occurs in a timeframe suitable to study the complete catalytic cycle, including the initial formation of the urea–urease complex.

### Using RuBpy–Urea in Urease Kinetic Assays

**Figure 4:**
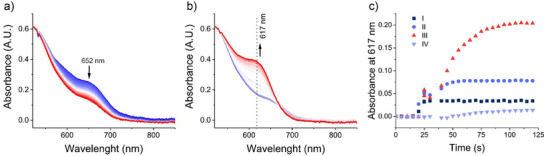
Photolysis of RuBpy–urea and urease activity assay. a) UV–vis absorbance spectroscopy monitoring of the photolysis of RuBpy–urea during irradiation with a diode laser (*λ* = 532 nm); the top blue line represents the initial spectrum and the bottom red line the last one over the course of 1 and a half hours of illumination. b) UV–vis absorbance spectroscopy of the reaction after photolysis and addition of bromothymol blue and jack bean urease; the blue represents the initial spectrum and the red the last one and c) increase of the absorbance at 617 nm at different conditions (I) after photolysis, 100 µL of 2.5 mM HEPES pH 6.5 buffer was added; (II) after photolysis 100 µL of 3 µM fluoride inhibited jack bean urease was added (2.5 mM HEPES pH 6.5 buffer); (III) after photolysis 100 µL of 3 µM jack bean urease was added (2.5 mM HEPES pH 6.5 buffer) and (IV) solution was maintained in the dark at 25 °C for 2 h, then 100 µL of 3 µM jack bean urease was added (2.5 mM HEPES pH 6.5 buffer).

With confirmation by FTIR that RuBpy–urea releases urea upon light irradiation, we decided to test whether RuBpy–urea could be used in an enzymatic assay. As a control, we performed urease kinetic assays in the presence of caged urea in the dark, to test whether it inhibits urease. These controls confirm there is no urease inhibition either in the presence of 10% of organic solvent or the caged urea (Figure S15). As the hydrolysis of urea by urease generates ammonia, increasing the reaction pH, a colorimetric pH assay was employed. We selected bromothymol blue (p*K*_a_ = 7), which changes from yellow to blue in a neutral solution, as a pH indicator. To enable visualization of the pH change, a low buffering capacity was employed (2.5 mM HEPES (4‐(2‐hydroxyethyl)piperazine‐1‐ethanesulfonic acid)), starting at pH 6.5. First, a solution of RuBpy–urea was photolyzed with a diode laser, which resulted in a decrease of the absorbance at 652 nm over the course of 2 h (Figure 4a). After this period, the pH indicator was added to the solution and the reaction was monitored at 617 nm (Figure 4b,c) following addition of either buffer, jack bean urease (JBU) or fluoride‐inhibited urease (Urease(F)). An increase of the band at 617 nm is observed due to the change of the spectrum of bromothymol blue, indicating an increase of the pH of the solution after addition of urease (Figure 4c and III). This increase in pH indicates that urease is active under these conditions, and that RuBpy–urea can be used to deliver urea to the enzyme in a controlled way. Control experiments with the addition of buffer in place of urease (Figure 4c and I) indicate that no pH change occurs due to the photolysis of the caged urea. As expected, fluoride‐inhibited urease reaches lower levels of activity in comparison to the uninhibited enzyme (Figure 4c and II). Lastly, an experiment in which no photolysis is performed, but urease is added (Figure 4c and IV) shows no pH increase, indicating that urease activity and the resulting pH increase does not occur in the dark, but instead requires photolysis of RuBpy–urea. These results support the utility of RuBpy–urea to probe urease activity and its potential for mechanistic studies of the enzyme.

### Detection of Urea–Urease Intermediates

Given that RuBpy–urea is photolabile and can deliver urea to urease, we decided to use this caged urea in a photocontrolled reaction with urease, probing the coordination with FTIR. The reaction intermediate of urea–urease in Urease(F) was detected by X‐ray crystallography.^[^ ^12^ ^]^ Therefore, the initial assays were performed using Urease(F) at room temperature to assess the feasibility of detecting intermediates (Figure S16). Photolysis of Urease(F) without RuBpy–urea revealed the emergence of bands at 1617 and 1541 cm⁻¹, along with a concomitant decrease of a band at 1588 cm⁻¹. These bands correspond to backbone amide I (1617 cm^−1^), amide II (1541 cm^‐^ ^1^, ^2^, ^3^, ^4^, ^5^), and side chain ν_as_ CO_2_^−^ (1588 cm^−1^) and could be related to an enzyme conformational change. Photolysis of ^12^C RuBpy–urea in the presence of Urease(F) revealed a band at 1636 cm⁻¹, which shifted to 1598 cm⁻¹ upon ^13^C labeling of the caged urea. The addition of fluoride to urease inhibits its activity, though there is still some active enzyme present (Figure S16) due to the limited fluoride concentration possible for these experiments. High fluoride concentrations cause precipitation of the caged urea, limiting its concentration to about 500 µM. Given the fluoride‐urease complex K_I_ of 1.02 ± 0.08 mM,^[^ ^66^ ^]^ the amount of inhibited urease for the FTIR experiments is only about 50% of the total enzyme concentration. Therefore, some of the photoreleased urea reacts with active enzyme and is ultimately converted to bicarbonate as evidenced by the band at 1636 cm⁻¹ (^12^C) attributed to the asymmetric stretch band of CO_2_^−^ of bicarbonate. Bands D and E of bicarbonate are also observed at 1363 cm^−1^ (ν_s_ CO_2_) and 1320 cm^−1^ (ρ COH).^[^ ^67^ ^]^ Interestingly, the FTIR of the photolysis in the presence of ^13^C‐RuBpy–urea presents bands at 1598 cm^−1^ (ν_as_ CO_2_), and ν_s_ CO_2_ at 1332 cm^−1^, corroborating to the formation of bicarbonate. Another band at 1507 cm⁻¹ was observed, whereas the spectrum in the presence of ^12^C caged urea has a band at 1588 cm⁻¹. These bands could be associated with the formation of the bridged O,N, urea–urease intermediate, but a nonambiguous assignment could not be obtained, due to the large isotopic shift (>80 cm^−1^). Attempts to decrease the intensity of the bicarbonate signal by decreasing the reaction temperature (15 °C) and the number of accumulated spectra (over 3 min) were unsuccessful. Thus, the detection of urea–urease intermediates, both for the inhibited and noninhibited enzyme, requires cryogenic conditions.

**Figure 5:**
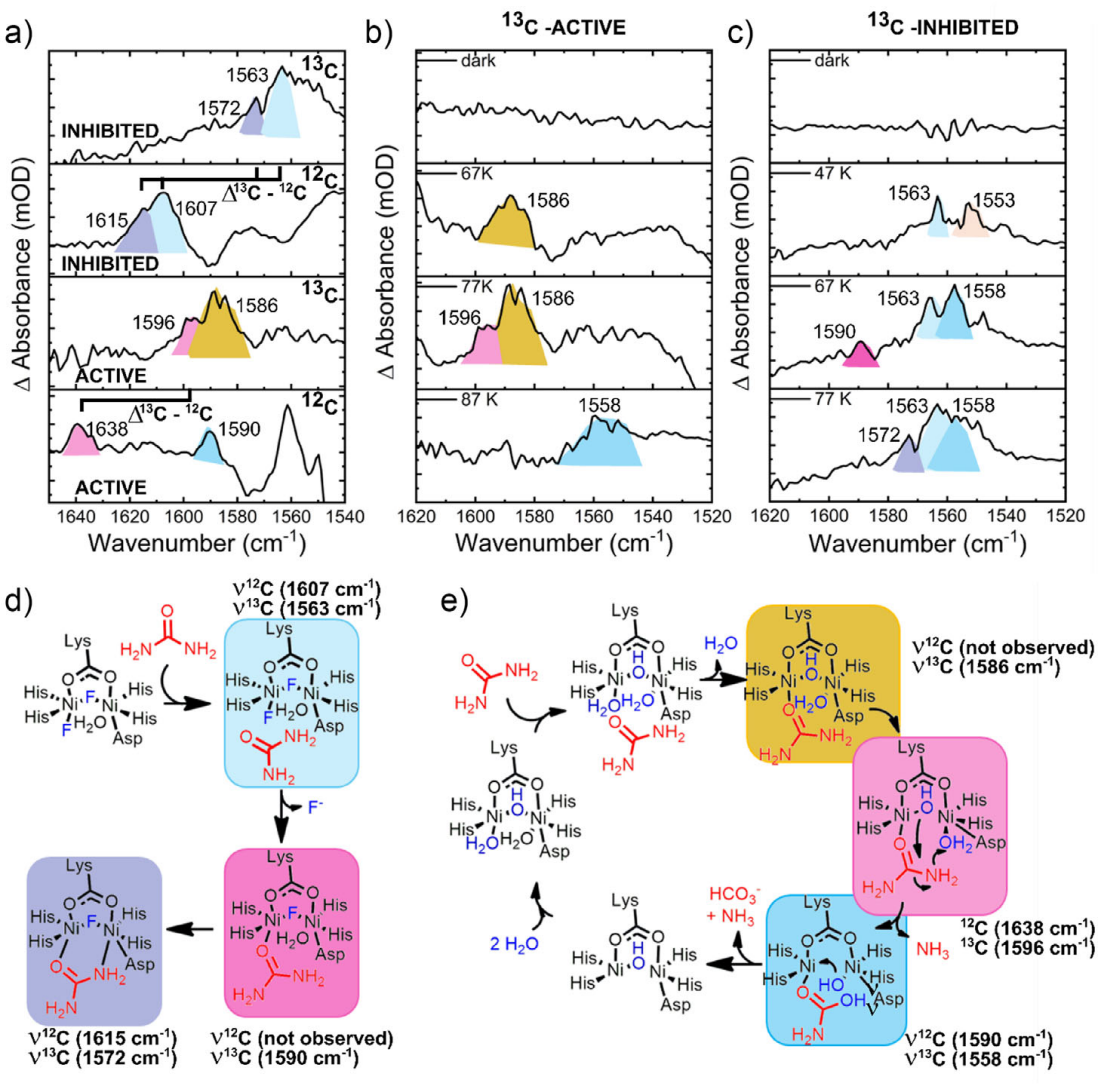
Cryo‐FTIR experiments after RuBpy–urea photolysis at pD 7.4. a) Isotope shift differential FTIR under cryogenic conditions (light‐minus‐dark) at 77 K for the active urease in the presence of RuBpy–urea. b) Differential FTIR under cryogenic conditions (light‐minus‐dark) at different temperatures for active urease in the presence of ^13^C RuBpy–urea. c) Differential FTIR under cryogenic conditions (light‐minus‐dark) at different temperatures for the fluoride inhibited urease in the presence of ^13^C RuBpy–urea. d) Scheme for the urea coordination mechanism involving fluoride‐inhibited urease. e) Scheme for the reaction of active urease with urea. Photolysis was performed with a 527 nm Nd:YAG laser (40 mW) for a set period.

Considering that the urea–urease intermediate of the inhibited enzyme is stable in the presence of fluoride and substrate, presenting a μ‐urea κ:O,κ:N coordination mode,^[^ ^12^ ^]^ we first tested fluoride‐inhibited urease, in order to assess the vibrational spectra associated to urea at the active site under this coordination mode at cryogenic conditions. In the absence of substrate urease does not present any spectroscopic change (Figure S17). As the solvent mixture for the cryogenic studies involved D_2_O buffer, glycerol‐D_6_ and acetone, to avoid any solvent peak overlap, all cryo‐FTIR assays shown in Figure 5b,c were performed with ^13^C‐caged urea and started at 47 K with increments of 10 K, reaching 87 K. The FTIR spectra in Figure 5 show the temperature and spectral regions where the major changes are observed during these experiments. (Figure S18 shows the broader temperature range and a sample of the raw FTIR spectral changes can be observed in Figure S19). At 47 K a *dark‐minus‐dark* spectrum was taken, and as seen in Figure 5c, no urea or urea–urease intermediate was observed. After irradiation of the fluoride inhibited urease sample with a Nd:YLF laser *(λ* = 527 nm, 50 mW/cm^2^) for 10 min∖, two peaks can be observed, one at 1553 and another at 1563 cm^−1^ (Figure 5c). The latter was assigned to the urea–urease(F) encounter complex, whereas the band at 1553 cm^−1^ could not be ascribed to a specific intermediate. It is possible it could be a νCN stretching band from the bridged coordinated urea. This might seem surprising, since the ability of urea to diffuse in a frozen glass at 47 K is unknown. However, jack bean urease has a PI (isoelectric point) of 5.9, resulting in a net negative charge of the urease surface, whereas the caged compound has a positive charge, therefore, there is an electrostatic interaction between the caged urea and urease, which increases the probability of having the photolyzed urea close to the active site. Upon increasing the temperature, the peak at 1563 cm^−1^ broadened and peaks at 1572 and 1590 cm^−1^ emerged. The 1590 cm ^−1^ peak disappeared at the highest temperatures (Figure 5c), indicating it could be a catalytic or unstable intermediate, whereas the 1572 cm^−1^ peak persisted at higher temperatures, suggesting it may be the urea–urease(F) intermediate. Broadening of the peaks might indicate interaction with water molecules^[^ ^68^ ^]^ as this increases solvent dynamics and changes the local electric field, causing a broadening of the νC═O vibration.^[^ ^69^ ^]^ In comparison to room temperature FTIR experiments (Figure S16), we can observe the band at 1572 cm^−1^ as a shoulder of the band at 1588 cm^−1^. Therefore, we assigned this band to the urea–urease(F) intermediate formation.

To verify the assignment of these peaks as urea–urease intermediates, we performed the cryo‐FTIR assays using ^12^C‐caged urea. As the FTIR spectrum of ^12^C‐urea has bands at higher frequencies than ^13^C‐urea and the acetone spectrum overlaps with these bands, we decided to perform the cryo‐FTIR using ^12^C‐RuBpy–urea in water–acetonitrile mixtures, but all other conditions were maintained. Under these conditions, we observed the emergence of bands at 1607and 1615 cm^−1^ due to the νCO bands of the ^12^C urea–urease intermediate, compared to the ^13^C bands at 1563 and 1572 cm^−1^, respectively, as shown in Figure 5a. The observed isotope shifts of 43–44 cm^−1^ are close to the expected value for a local oscillator model and confirm the assignment of these bands to coordinated urea.

To further assist in the assignment of the observed peaks, we also compared the frequencies above to those reported for urea coordination to urease mimics (Table 1).^[^ ^38^ ^]^ These complexes used ^12^C urea and we must take into consideration the differing carbon isotopes, which results in a correction to the reported wavenumbers from ^12^C to ^13^C urea of approximately −40 cm^−1^. For instance, the μ‐urea κ:O,κ:N coordination mode observed at 1614 cm^−1^ (νCO)^[^ ^38^ ^]^ should be expected at 1572 cm^−1^ (νCO) for a ^13^C‐labeled urea.

**Table 1:** Comparison between FT‐IR bands of observed for different urea coordination modes in inorganic models, the calculated corrected ^13^C band and the observed bands of the inhibited (urease(F)) and noninhibited urease after urea photorelease.

Examples	νCO (cm^−1^)	^13^C (cm^−1^)^a^	νCN (cm^−1^)	^13^C (cm^−1^)^a^	Urease(F)‐ ^12^C urea	Urease(F)‐ ^13^C urea	Urease‐ ^12^C urea	Urease‐ ^13^C urea
**^12^C μ‐urea κ:O,κ:N ^[^** **38** **^]^**	1614	1572	1572	1532	1615 (νCO) 1607 (νCO)	1572 (νCO) 1563 (νCO)		
**^12^C μ‐carbamate κ:O,κ:O ^[^** **38** **^]^**	1590	1550	1571	1531			1590 (νCO)	
**^12^C κ‐O urea ^[^** **37** **^]^**	1642	1602						
**^12^C κ‐O urea ^[^** **71** **^]^**	1640	1600				1590 (νCO)	1638 (νCO)	1586(νCO) 1596 (νCO)
**^12^C κ‐O urea ^[^** **71** **^]^**	1672	1632						
**^12^C κ‐O urea ^[^** **70** **^]^**	1663	1623 1569	1576	1536				
**^12^C κ‐O urea ^[^** **70** **^]^**	1661	1621 1597	1611 1578	1571 1538				
**^12^C κ−O urea ^[^** **39** **^]^**	1669	1629	1544	1504				

Coordination of the carbonyl (κ‐O urea), by contrast, is expected at higher frequency such as 1654 cm^−1^ (ν CO), 1640 cm^−1^ (ν CO), 1663 cm^−1^ (ν CO), 1609 cm^−1^ (ν CN), 1669 cm^−1^ (ν CO), and 1620 cm^−1^ (ν CN), which upon ^13^C correction should be expected at 1612, 1598, 1621, and 1627 cm^−1^.^[^ ^37^, ^39^, ^70^, ^71^ ^]^ These bands are summarized in Table 1. Therefore, as described above, the ^12^C νCO band of the bridged coordination mode in urea–urease(F) was observed at 1607 cm^−1^, whereas ^13^C urea–urease(F) intermediates observed after RuBpy–urea photolysis appeared at 1563, 1572, and 1590 cm^−1^ upon an increase in temperature. Interestingly, this is in agreement with the expected isotopic shift, where the unlabeled urea–urease(F) intermediate was observed at 1615 and 1607 cm^−1^ (Figure 5a). Therefore, we ascribe the 1572 cm^−1^ to the bridged bidentate mode of coordination of urea, whereas the less intense band at 1590 cm^−1^ was assigned to the κ‐O urea coordination mode.

We performed a similar isotope‐edited cryo‐FTIR experiment with the uninhibited urease (Figure 5b). In these experiments at 47 K, no band was observed, even after irradiation. However, at 57 K a band at 1586 cm^−1^ emerged. This band broadened at 67 K, perhaps due to loss of the coordinated water. At 77 K, a new band at 1596 cm^−1^ emerged, which upon further heating to 87 K, disappeared, indicating that it could be the active urea–urease intermediate. The effective absence of a peak at 1572 cm^−1^ (Figure 5b) suggests that the assembly of possible urea–urease intermediates is different than for the urea–urease(F) intermediate. For instance, the uninhibited urea–urease cryo‐FTIR spectrum is dominated by bands associated with the κ‐O urea coordination mode around 1590 cm^−1^, whereas the fluoride‐inhibited urea–urease spectrum primarily displays the peak associated with the μ‐urea κ:O,κ:N coordination mode. The use of nonlabeled caged urea revealed bands at 1638 and 1590 cm^−1^ (Figure 5a). These bands correspond to the expected isotopic shift of the ^13^C bands at 1596 and 1558 cm^−1^, respectively, as shown in Figure 5a.

Another difference between data for the active and inhibited enzyme complexes is the presence of a band at 1586 cm^−1^ associated with uninhibited urease. This band could be the pre‐equilibrium urea–urease encounter complex, which upon heating, enables urea coordination and allows the formation of the intermediate urea–urease, with a band at 1596 cm^−1^. The attack of the carbonyl enables the formation of carbamate with a spectral band at 1558 cm^−1^. Interestingly, in the inhibited version of the enzyme, the encounter complex (1586 cm^−1^) was not observed. This difference could be due to the distinct starting active sites and their corresponding coordination of urea to Ni(1) and attack of the carbonyl by the bridged hydroxide to form products. Regeneration of the active site is then enabled by the water coordinated to Ni(2).

Based on these observations, we propose mechanisms for the inhibited and active enzyme (Figures 5d,e, respectively) and associate the observed IR frequencies to different intermediates. For instance, once urea is photolyzed from the caged urea, it forms an encounter complex with urease. 1) In the active enzyme, this is followed by O‐coordination of urea to Ni(1) and attack of the carbonyl by the bridged hydroxide to form products. Regeneration of the active site is then enabled by the water coordinated to Ni(2). 2) In the inhibited urease, urea coordinates in an O,N bridged mode, and since it is inhibited, no other reaction occurs.

**Figure 6:**
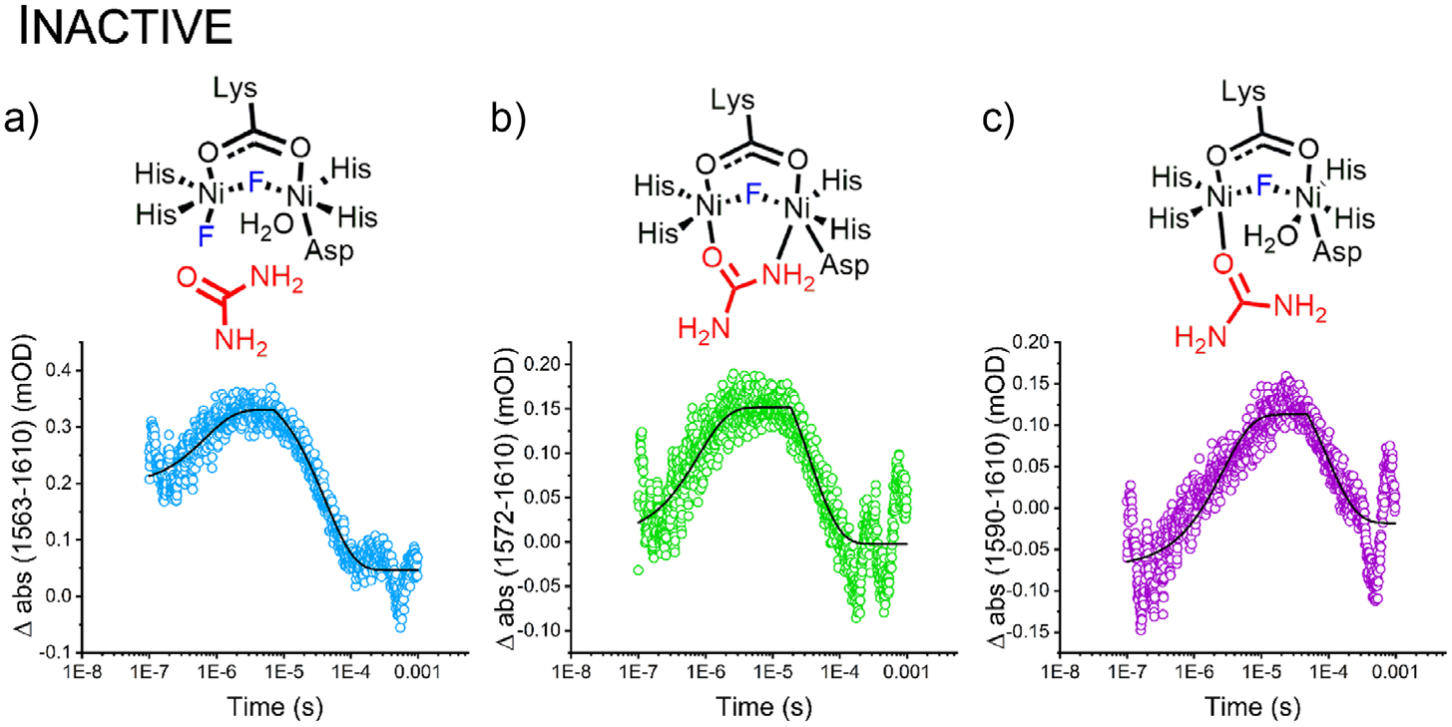
Transient IR absorption experiment of the inactive (fluoride inhibited) urease in the presence of RuBipy‐^13^C urea at room temperature. a) Transient absorption detected at 1563 cm^−1^ corresponding to the encounter complex of inactive urease and urea, b) absorption detected at 1572 cm^−1^ corresponding to the μ‐urea  κ:O,κ:N coordination mode in the inhibited urease, C) absorption detected at 1590 cm^−1^ corresponding to the urea  κ:O coordination mode in the inhibited urease. A single laser pulse (Spectra Physics GCR‐3 Nd:YAG, 532 nm; ca 100–500 µJ; 10 ns duration) initiated the reaction.

To validate these assignments, time‐resolved IR experiments were performed for both active and inhibited enzyme in solution at room temperature (Figure 6). For these experiments, a Q‐switched Nd:YAG laser pulse (532 nm, 10 ns) was used to photolyze the cage and release free urea within 80 ns as shown in Figure 3. The reaction of free urea with the enzyme was probed by transient IR absorbance at specific frequencies corresponding to the peaks observed in the cryo‐FTIR experiments. In the case of the fluoride‐inhibited urease (Figure 6a–c), the bands at 1563, 1572, and 1590 cm^−1^ were monitored and a solvent reference transient (1610 cm^−1^) was subtracted to obtain the enzyme transients shown in Figure 6. The first two bands have similar rise times at the 500–600 ns timeframe, and decay times between 40 and 50 µs, respectively. As observed in the cryo‐FTIR data, a band at 1590 cm^−1^ appears at higher temperatures, and interestingly this band had a better fit using a double exponential, resulting in two different rise times. The first rise occurs at ≈500 ns, in agreement with the formation of the κ‐O urea coordination mode in the same timescale of the formation of the encounter complex (1563 cm^−1^) and the bridged coordination mode (band at 1572 cm^−1^). The second rise occurs at a later timescale (8 µs), suggesting that the κ‐O urea and μ‐urea κ:O,κ:N coordination modes are in equilibrium. Interestingly, the 1590 cm^‐^ ^1^, ^2^, ^3^, ^4^, ^5^ band decays at 95 µs. Owing to its emergence at later timescales, we propose that this is a reactive state formed later in the reaction probably due to an equilibrium shift of the bound urea and uncoordinated water. One might expect that the reaction should stop at the μ‐urea κ:O,κ:N stage, according to Figure 5d. However, fluoride is a pseudo‐uncompetitive inhibitor of urease that forms a stable inhibited complex with the enzyme in the presence of urea, but its stability diminishes in the absence of the substrate or at low substrate concentrations. Under such conditions, fluoride can dissociate, regenerating the reactive enzyme.^[^ ^72^ ^]^ Therefore, considering that the caged urea is 4 times less concentrated than urease and that the quantum yield of uncaging is low (0.13%), we are delivering far below the 1:1 stoichiometry to maintain the enzyme in the inhibited mode and the decay observed for these bands is not surprising.

Contrasting the inhibited enzyme assay, time‐resolved IR experiments with the uninhibited urease produced weak transient absorbance changes with a low signal/noise (S/N) ratio (Figure S20). The TRIR transients for the active urease were also obtained after the subtraction of a solvent reference transient at a frequency where only the solvent absorbs (see raw transients at Figure S21). Small absorbance changes above the S/N of the experiments are observed for the band at 1596 cm^−1^, which corresponds to the active urea–urease intermediate and for the band at 1558 cm^−1^, corresponding to product formation. These results are puzzling considering that we used the same concentration of urease and caged urea in both experiments (inhibited and uninhibited). The faster kinetics of the active enzyme could prevent the intermediate states from building up appreciably on the timescale of diffusion of substrate into the enzyme (≈500 ns based on Figure 6a–c). For this interpretation to be valid, the formation and decay of the intermediate states would have to be significantly faster than 500 ns. The kinetics observed for the intermediate formation (200 ns rise and 376 µs decay) are consistent with the profile observed for the inhibited enzyme. Furthermore, carbamate product formation occurs with a rise time of 1.8 ms, (Figure S20) corresponding to completion of one full reaction cycle, in agreement with a *k*_cat_ of order 10^3^–10^4^ s^−1^.^[^ ^81^ ^]^ These lifetimes indicate that the rate limiting step would have to be the product release to yield the overall lifetime (≈1 ms) observed for enzyme turnover.

### Implications to the Competition Mechanism

The competition mechanism hypothesizes that there is a competition between catalysis (*k*_cat_) and water exchange rate (*k*_ex_) in metalloenzymes that can impact the enzyme mechanism.^[^ ^73^, ^74^ ^]^ Enzymes that have high *k*_ex_ and low *k*_cat_ (*k*_cat/_ *k*_ex _< 1) predominantly coordinate their substrate to the metal ion, whereas the inverse (*k*_cat_/*k*_ex_ > 1) corresponds to employment of the hydrated‐metal ions during catalysis.

To understand the competition mechanism in urease, it is necessary to divide the reaction into different steps (Figure 5e). For instance, the proposed mechanism involves urea coordination to Ni(1) through the carbonyl oxygen (κ‐O) followed by dehydration of Ni(2)^[^ ^75^ ^]^ and coordination of urea in a κ:O,κ:N coordination mode.^[^ ^24^, ^76^ ^]^ The last step of the mechanism is the catalytic process, in which the bridged hydroxyl attacks urea carbonyl to form carbamate. However, if there is competition between the hydroxyl attack and the urea coordination in a bridging mode, a κ‐O coordination of urea would most probably be favored, reducing reaction steps, as shown in Figure 5e. Interestingly, the competition mechanism proposes that at lower pHs, the bridged coordination mode of urea would be favored due to the decrease in *k*_cat_.^[^ ^74^ ^]^ To verify if this possibility might be occurring, we performed cryo‐FTIR of urease at pD 5.5 (50 mM HEPES buffer). Remarkably, these experiments yielded the encounter complex band at 1586 cm^−1^ at all temperatures, with the exception of temperature 47 K (Figure S22). Moreover, both the κ‐O coordinated urea (1596 cm^−1^) and the μ‐urea κ:O,κ:N coordination mode (1572 cm^−1^) were observed at 57 and 67 K. The band corresponding to carbamate (1558 cm^−1^) is more evident at 67 K. To verify if the intensities of the observed bands were significant, we performed an additional experiment at pD 5.5, but now at a constant temperature (77 K). At this temperature the reaction can proceed slowly, and we monitored the formation of the reaction intermediates over time (Figure S23). In this experiment we observed that the band relative to the urea–urease encounter complex (1586 cm^−1^) increased over time, ranging from noise levels to 0.7 mOD (yellow column, Figure S23B).The bands relative to the coordinated urea (κ‐O coordinated urea,1596 cm^−1^ and μ‐urea κ:O,κ:N coordination mode, 1572 cm^−1^) are more intense after 70 min of reaction, reaching intensities of 1.5 and 0.7 mOD, respectively. The band relative to the product formation (1558 cm^−1^) also reaches 1.5 mOD of absorption at the end of the experiment (70 min). As a control, we also monitored the band at 1563 cm^−1^, which is only present in the fluoride inhibited urease and as in this experiment an active urease was used, we were not expecting any changes over time. In fact, this band does not increase over time, remaining at noise levels throughout the experiment (light blue bars, Figure S23B). An interesting feature observed in this experiment was the presence of a high intensity band at 1540 cm^−1^, reaching intensities of almost 3 mOD. This band was attributed to free carbamate.^[^ ^77^ ^]^ These results indicate that depending on the *k*_cat_ of the reaction, the μ‐urea κ:O,κ:N coordination mode increases its probability of occurring. For instance, at pD 5.5 the ratio between κ‐O coordinated urea (1596 cm^−1^) and μ‐urea κ:O,κ:N coordination mode (1572 cm^−1^) is about 2, whereas at pD 7.4 this ratio is almost 13. Thus, most probably there is competition between coordination modes depending on the reaction environment and thus we observe the presence of a higher content of bridged urea at a lower pH.

Considering that *k*_cat_ influences the ratio of coordination modes observed in urease catalysis, one can attempt to expand the competition hypothesis to explain the metal selectivity of urease.^[^ ^78^, ^79^, ^80^ ^]^ Taking urease from jack bean as an example, its *k*_cat_/*k*_ex_ can be used as an attempt to understand the higher activity of nickel‐ureases. For instance, *k*_ex_ of [Ni(H_2_O)_6_]^2+^ is in the same magnitude (2.34x10^4^ s^−1^)^[^ ^81^ ^]^ of jack bean urease *k*_cat_ (3.2x10^4^ s^−1^),^[^ ^82^ ^]^ resulting in *k*_cat_/*k*_ex_ of 0.73. However, iron(II)‐containing ureases^[^ ^83^ ^]^ have a lower *k*_cat_/*K*_M_,^[^ ^78^ ^]^ which indicate either a lower *k*_cat_ or a much higher *K*_M_. Assuming that ureases have similar *K*_M_, we can estimate that *k*_cat_ value of these enzymes are lower than for nickel‐containing ureases. Using the *k*_ex_ of [Fe(H_2_O)_6_]^2+^ (4.4 × 10^6^ s^−1^) we would expect a value much lower than one for *k*_cat_/*k*_ex_ of iron(II)‐containing ureases, resulting in more bridged urea during catalysis. This disparity could be attributed to competition between the dehydration of Ni(2) and catalysis in urease. The most catalytically active species is κ‐O coordinated urea, which is more favored in nickel‐containing ureases. In contrast, iron‐containing ureases may preferentially stabilize the less active κ:O,κ:N coordination mode, resulting in reduced catalytic efficiency. The methodology developed in this work can be used to test this hypothesis.

## Conclusion

Using a photolabile caged urea and cryo‐FTIR, we were able to identify important bands related to the catalysis of urease. Through analysis of these bands and by comparison to the fluoride‐inhibited enzyme, we demonstrated a dissimilarity between systems, indicating that fluoride inhibition changes the coordination mode of urea. The most probable cause of this change is competition between *k*_cat_ and *k*_ex_, which enables pre‐dominantly bridged coordination of urea in the inhibited urease. Transient infrared absorbance revealed that photorelease of urea and its subsequent coordination to the enzyme occur in the ns timeframe. Uninhibited urease was shown to pass from an encounter complex to a κ‐O urea, which was followed by the formation of carbamate at later periods (ms). This study sheds light on the proposed competition between *k*_cat_ and *k*_ex_ in the urease mechanism, and we believe it could be extended to other metalloenzymes. Thus, we demonstrate the importance of photocaged substrates and cryo‐trapping of intermediates without using inhibitors or mutations that might slow down the catalytic step and change the mechanism of a metalloenzyme.

## Supporting Information

Supporting Information contains UV–vis, HRMS, ^1^H, ^13^C, ^31^P, and ^15 ^N NMR of RuBi‐Urea. FTIR spectra of urea, ^15^N‐urea and ^13^C‐urea. Cryo‐FTIR of urease at pD 5.5.

## Conflict of Interests

The authors declare no conflict of interest.

## Supporting information



Supporting Information

## Data Availability

The data that support the findings of this study are available in the Supporting Information of this article.
