# Supplementary material for: Photocontrolled Release of Urea Enables the Detection of Urea–Urease Intermediates by Cryo‐FTIR
Source: Angew Chem Int Ed Engl. 2025 Jul 25;64(36):e202504332. doi: 10.1002/anie.202504332 (PMC12402847; doi:10.1002/anie.202504332)
Supplement: Supplementary file 1 — Supporting Information [file ANIE-64-e202504332-s001.docx]

**Photo-controlled release of urea enables the detection of urea-urease intermediates by cryo-FTIR**

Caterina G.C. Marques Netto ^[a],[b]^ ^†^*, Sarah Bell ^[b]^, Caio B. Castro ^[a]^, Pedro Henrique Machado^[a]^, Vatsal Purohit^[b]^, Katherine M. Davis^[b]^, Ana Paula L. Batista^[a]^, R. Brian Dyer ^[b]^ **

**Contents:**

[1. Experimental protocols 2](#_Toc187767984)

[1.1 Electronic Spectroscopy at the UV-Vis region 2](#_Toc187767985)

[1.2 Photolysis assays monitored by UV-Vis Spectroscopy 2](#_Toc187767986)

[1.3 Urease kinetic assays after photolysis 2](#_Toc187767987)

[1.4 NMR experiments 3](#_Toc187767988)

[1.5 High Resolution Mass Spectrometry 3](#_Toc187767989)

[1.6 Microanalysis 3](#_Toc187767990)

[1.7 Caged Urea (RuBipy-Urea) synthesis: 3](#_Toc187767991)

[1.8 FTIR Sample Preparation : 4](#_Toc187767992)

[1.9 FTIR Phototitration at Room Temperature: 4](#_Toc187767993)

[1.10 FTIR Phototitration at 70 K of RuBpy-urea: 5](#_Toc187767994)

[1.11 Preparation of urease 5](#_Toc187767995)

[1.12 FTIR Phototitration at low temperature of RuBpy-urea in the presence of urease: 6](#_Toc187767996)

[1.13 Time-Resolved Infrared (TRIR) Spectroscopy : 6](#_Toc187767997)

[1.14 Uncaging of urea and urease assays: 7](#_Toc187767998)

[1.15 Computational method 8](#_Toc187767999)

[2.Spectra 8](#_Toc187768000)

[3. References 21](#_Toc187768001)

1. Experimental protocols

All manipulations of the caged urea were performed in a dark room. All manipulations were performed using a red light to enable the visualization of the samples/experiments.

## 1.1 Electronic Spectroscopy at the UV-Vis region

UV-Vis spectra were recorded using an Ocean Optics QE65000 spectrometer with a xenon lamp source. The molar absorptivity experiments of RuBpy-urea were recorded using acetone as a solvent.

## 1.2 Photolysis assays monitored by UV-Vis Spectroscopy

Photolysis of RuBpy-urea were performed using an Ocean Optics QE65000 spectrometer with a xenon lamp source. For the assays, 100 μL of a stock solution of RuBpy-urea (1 mg dissolved into 500 μL of acetone) and 1 mL of HEPES buffer pH 6.5 (2.5mM) were mixed in a quartz cuvette. The cuvette was placed on a cuvette holder with temperature control at 25°C and constant stirring of the solution using a magnetic stirrer placed at the side of the cuvette. The cuvette was irradiated using a photo diode laser (λ = 532nm, 4mW) for 1.5 hours. The band at 652 nm was monitored until it dropped its absorbance to half of its value (initial absorption: 0.245; final absorption: 0.122). A control experiment was performed where the solution was maintained in the dark at 25 ºC for 2 hours.

## 1.3 Urease kinetic assays after photolysis

After photolysis an urease assay was performed to verify if urea was actually released. For that, we monitored the increase of the absorbance at 617 nm due to bromothymol blue as a pH indicator. In these assays, a control adding only the urease buffer was performed, where 100 μL of 2.5 mM HEPES pH 6.5 buffer was added to the cuvette. In another experiment, after photolysis, 100 μL of 3 μM of either active or inhibited jack bean urease was added (2.5 mM HEPES pH 6.5 buffer).

## 1.4 NMR experiments

^1^H, ^15^N, ^31^P and ^13^C NMR of RuBpy-urea were recorded in a 600MHz Bruker BioSpin GmbH using acetone-d6 as a solvent. The ^1^H NMR stability test of RuBpy-urea was performed using a 50% acetone, 50% D_2_O medium. The NMR tube was left in the dark during this analysis and the spectra were recorded in a 400 MHz BRUKER ARX 9.4 T

## 1.5 High Resolution Mass Spectrometry

The RuBpy-urea was dissolved in acetone and ionized by electrospray (ESI) and analyzed at the Mass Spectroscopy Emory Core, employing a Thermo Exactive Plus equipment

## 1.6 Microanalysis

The microanalysis of RuBpy-urea was performed by the Analytical Central from the Department of Chemistry at UFSCar using an EAGER 200 CE equipment.

1.7 Caged Urea (RuBpy-Urea) synthesis: A 1-neck 25 mL round bottom flask was charged with 300 mg of [Ru(bpy)_2_PPh_3_Cl]PF_6_ (0.35 mmol, 1 eq.) ^1^, 200 mg of NaCl (3.5 mmol, 10 eq.), 530 mg of urea (8.8 mmol, 25 eq.), 10 mL of N_2_-purged ethanol and 1mL of 2M NaOH solution (N_2_ purged). The reaction was heated to 78 ºC for 3 hours under reflux. After that period, 3mL of 1M HCl was added to the reaction and the content was centrifuged at 6000 rpm for 5 minutes. The solid was discarded and to the solution was added a saturated aqueous solution of KPF_6_ (1 mL), followed by the addition of water (~25 mL) until complete precipitation of a brown solid. The solution was centrifuged at 6000 rpm for 5 minutes and the solid was discarded. The solution was added to a separating funnel, and it was extracted with CHCl_3_ (15x 10 mL). The first two extractions presented a brown color and were discarded, whereas the subsequent ones that presented a purple color were combined in an Erlenmeyer flask, dried with MgSO_4_, filtered and the solvent was removed in a rotatory evaporator. The sample was stored away from light exposure in an -80 ºC freezer. Yield: 5%, 10 mg.

^1^H NMR (600 MHz, CDCl_3_, δ ppm): δ10.16 (d, 1H), 9.28 (d, 1H), 8.58 (d, 2H), 8.70 (dd, 2H), 8.43 (d, 2H), 8.37 (d, 1H), 8.12 (m, 3H), 7.75(m, 3H), 7.15 ddd (J=7.2, 5.8, 1.2 Hz, 1H), 7.03 ddd (J=7.3, 5.8, 1.3 Hz, 1H), 6.41(s, 2H), 5.73 (s, 2H), 5.12 (s, 6H).

^31^P NMR (600 MHz, CDCl_3_, δ ppm): -144.3 (q)

^13^C NMR (600 MHz, CDCl_3_, δ ppm): δ 167.1, 160.9, 160.5, 159.5, 159.0, 158.5, 154;1, 153.87, 153.01, 151.63, 136.2, 135.8, 135.1, 134.3, 126.1, 126.0, 125.8, 125.2, 123.1, 122.9, 122.95

HRMS: m/z 509.04263 (found), 509.04305 (calculated for C_21_H_20_ClN_6_ORu)

Anal. Calcd for C_29_H_44_ClF_6_N_10_O_7_PRu (RuBi-Urea[C_21_H_20_ClF_6_N_6_OPRu]•2CH_6_O•2H_2_O •2CONH_4_): C 37.61; H 4.79; N 15.12. Found: C: 37.45; H: 4.82; N 14.78

## **1.8 FTIR Sample Preparation :**

A microtube containing caged urea was allowed to thaw prior to sample preparation. Then, 5 μL of acetone and 15μL of 50 mM Tris-DCl pD 7.3 were added to the tube for the dissolution of the caged urea. A fresh solution was prepared for each of the assays: phototitration of the caged urea and transient absorption.

1.9 FTIR Phototitration at Room Temperature: FTIR spectra were recorded using a modified Varian 660 FT-IR spectrometer. For transmission FTIR, the IR beam is sent to a purged external sample chamber to an external detector. The sample was loaded into a sample cell consisting of a 50 μm PTFE spacer between two CaF_2_ windows (Harrick) held in a copper housing. The solution containing the caged urea was loaded into the cell using a 20 μL Hamilton syringe. UV-Vis spectroscopy of the cell revealed an absorption at 527 nm of 0.45.

All transmission FTIR spectra are the average of 2048 scans. All spectra are ratioed to a first spectrum in the dark, prior to irradiation. Before irradiation, a second set of “dark” spectra (2048 scans) were collected and the difference between both dark spectra (ΔA) was calculated as dark-minus-dark. The sample was illuminated at 527 nm with a Nd:YLF laser (CrystaLaser) for a set length of time. Following illumination, spectra were obtained and the process was repeated. Difference spectra (ΔA) were calculated by ratioing against the previous dark spectrum.

1.10 FTIR Phototitration at 70 K of RuBpy-urea: Low-temperature spectra were collected on a Bio-Rad FTS60A/896 Step-Scan FTIR spectrometer. Samples were mounted in a closed cycle Helium cooled APD Cryogenics cryostat and maintained at 70 K. Reference spectra consisting of “dark” spectra (2048 scans) were collected at 4 cm^-1^ resolution immediately before photolysis. Before irradiation, another set of “dark” spectra (2048 scans) were collected and the difference between both dark spectra (ΔA) was calculated as dark-minus-dark. The sample was then photolyzed with a 527 nm Nd:YAG laser (40 mW) for a set period. Difference spectra (ΔA) were calculated by ratioing against the previous dark spectrum.

## 1.11 Preparation of urease

*Jack Bean* urease was obtained from Sigma-Aldrich and was prepared as follows: 200 mg of urease was resuspended in a buffer containing 50 mM HEPES, 5 mM EDTA and 5 mM β-mercaptoethanol pH 6.5. The solution was centrifuged at 277 K for 30 min at 10 000 rev min^-1^. The supernatant was concentrated and loaded onto a Sephacryl S-300 gel-filtration column (GE Healthcare Lifesciences) which was pre-equilibrated in the same buffer and the peak containing active urease fractions was collected and pooled. After that, urease was buffer exchanged using a 50KDa centricon filter. After 5 exchanges, the solution was lyophilized. After lyophilization, 500 μL of D_2_O were added to it to resuspend it, and we performed the lyophilization once more. This procedure was repeated 3 times. Samples were stored lyophilized and frozen until its use. Prior to their use, 500 μL of D_2_O was added and using a 50 kDa centricon filter we concentrated the solution until reach the desired concentration (1mM for the FT-IR experiments).

## 1.12 FTIR Phototitration at low temperature of RuBpy-urea in the presence of urease:

Low-temperature spectra were collected on a Bio-Rad FTS60A/896 Step-Scan FTIR spectrometer.

In the dark, 8 μL of the ^13^C-caged urea solution (46 mM) was mixed with 8μL of deuterated glycerol and 16 μL of the *Jack Bean* urease solution (≈8 mM). Then, the sample was loaded into a sample cell consisting of a 50 μm PTFE spacer between two CaF_2_ windows (Harrick) held in a copper housing. Immediately after that, the sample was mounted in a closed cycle Helium cooled APD Cryogenics cryostat and maintained at 47K. The water-glycerol solution forms a glass at this temperature that is transparent to the IR and to the laser beam used for photolysis.

References of “dark” spectra (2048 scans) were collected at 4 cm^-1^ resolution immediately before photolysis. Before irradiation, another set of “dark” spectra (2048 scans) were collected and the difference between both dark spectra (ΔA) was calculated as dark-minus-dark. The sample was then photolyzed with a 527 nm Nd:YAG laser (40 mW) for a 20 minutes. Then, the temperature was set to 57, 67, 77 and 87 K. Difference spectra (ΔA) were calculated by rationing against the previous dark spectrum. As the increase in temperature caused the baseline to drift, we baseline corrected the difference spectra by subtracting a straight line.

1.13 Time-Resolved Infrared (TRIR) Spectroscopy : TRIR spectroscopy employed a ^13^C RuBi-urea solution to measure the kinetics of photorelease of the caged urea and were performed in solution at room temperature. A single laser pulse (Spectra Physics GCR-3 Nd:YAG, 532 nm; ca 100-500 μJ; 10 ns duration) initiated the reaction. Laser intensity was attenuated to achieve photorelease while minimizing temperature-jump and cavitation artifacts. Transient absorbance traces were collected with single laser pulses focused to a 500 µm diameter spot (50 mJ/cm^2^) in all experiments. Each transient was collected from a fresh sample volume by shifting the sample position before the laser shot (diffusion from an illuminated spot through the total volume of the sample is slow). Multiple laser shots (100-1000) were collected and averaged. The IR transients were fit using Origin software single-exponential fit functions for a determined range of the spectrum (rise or decay) after correcting the spectrum by subtracting a reference at a region what does not have signals. Figure 6C was fit with a double exponential, as its shape justified it.

In the case of urease containing experiments, 25 μL of 1mM *Jack Bean* urease sample in Tris-HCl buffer (50 mM, pH 7.5) was mixed with 8 μL of an acetone solution containing 5 mg of RuBpy-urea. This mixture was loaded into a sample cell consisting of a 50 μm PTFE spacer between two CaF_2_ windows (Harrick) held in a copper housing.

1.14 Uncaging of urea and urease assays: For this assay, the increase of the band at 617 nm of the pH indicator bromothymol blue was monitored over time on an Ocean Optics QE65000 spectrometer with a Xenon lamp source. A semi-micro quartz cuvette of 1 cm pathlength was employed. A 1.1 mL solution of caged urea in HEPES buffer (2.5 mM, pH 6.5) with 10% acetone was prepared to achieve a concentration of 14 mM. This solution was irradiated with a diode laser (λ = 532 nm, 4.6 mW) for 2 hours. After that period, 5 μL of a saturated solution of bromothymol blue and 100 μL of a 3 μM *Jack Bean* urease solution (HEPES buffer (2.5 mM, pH 6.5)) were added and the absorption at 617 nm was monitored.

Controls of this assay were performed by 1) adding 100 μL of fluoride inhibited *Jack Bean* urease (3 μM urease and 1mM fluoride), 2) adding 100 μL of HEPES buffer (2.5 mM, pH 6.5)) and 3) in the dark for 2 hours, then adding 100 μL of a 3 μM *Jack Bean* urease solution (HEPES buffer (2.5 mM, pH 6.5)).

## 1.15 Computational method

Density functional theory (DFT) calculations were carried out using ORCA, ^2^ version 5.0.4. Optimized geometries were obtained using the B3LYP ^3-5^ functional and the def2-TZVP ^6, 7^ basis set, as these have been successfully applied to explore structural and electronic proper-ties of related systems. ^8-11^ The D3(BJ) ^12, 13^ dispersion correction was also applied during these optimizations. Furthermore, the effect of bulk solvent was considered by employing the conductor-like polarizable continuum model ^14^ (CPCM). The vibrational analysis of all structures was performed to confirm them as a minimum in the explored potential energy surface (PES).

**o**

# 2.Spectra

Figure S1. ^1^H NMR of RuBpy-urea dissolved in acetone-d6.

Figure S2. ^13^C NMR of RuBpy-urea dissolved in acetone-d6.

Figure S3. ^31^P NMR of RuBpy-urea dissolved in acetone d6.


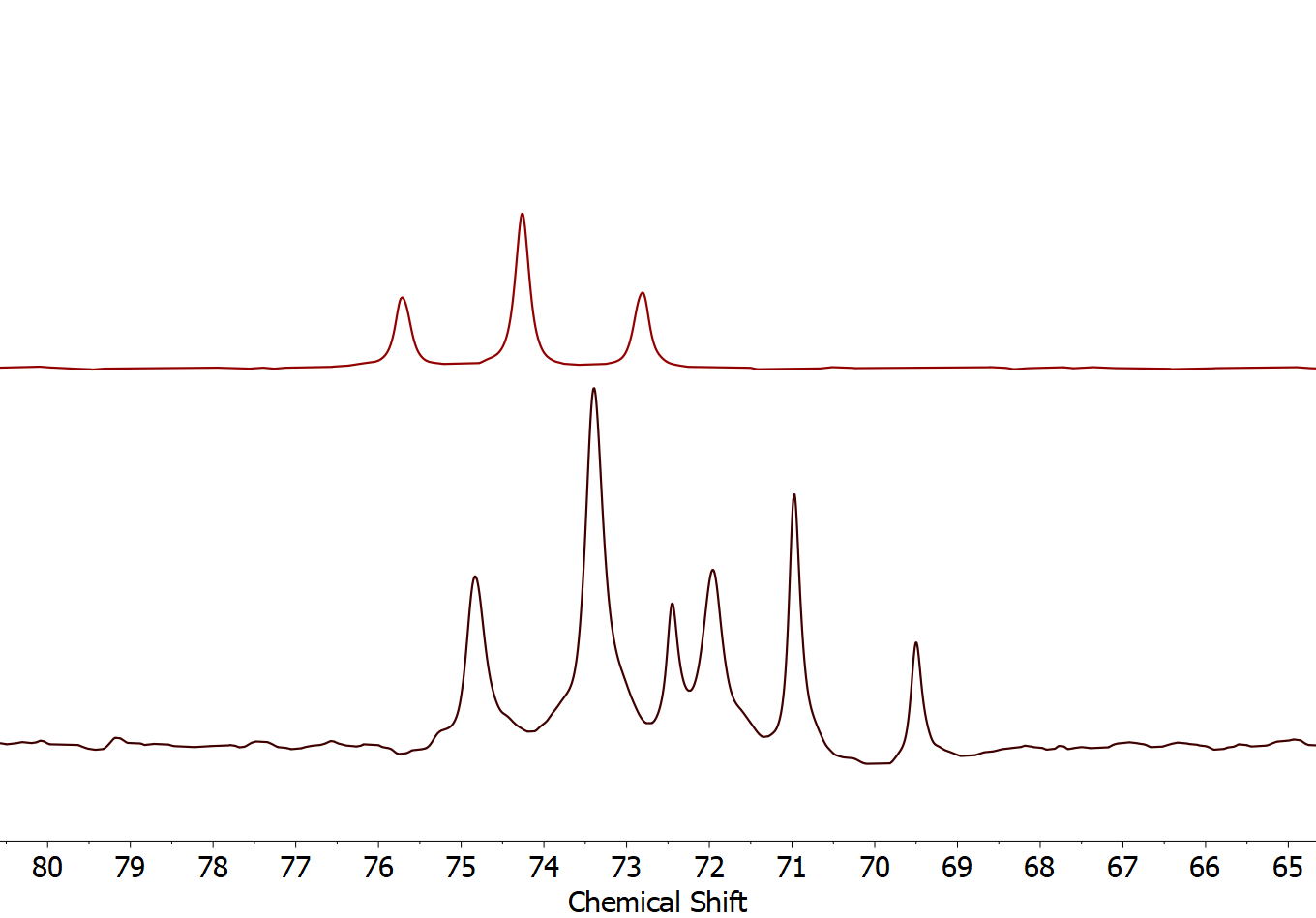


Figure S4. ^15^N NMR of urea (top) and RuBpy-urea (bottom) dissolved in CDCl_3_.


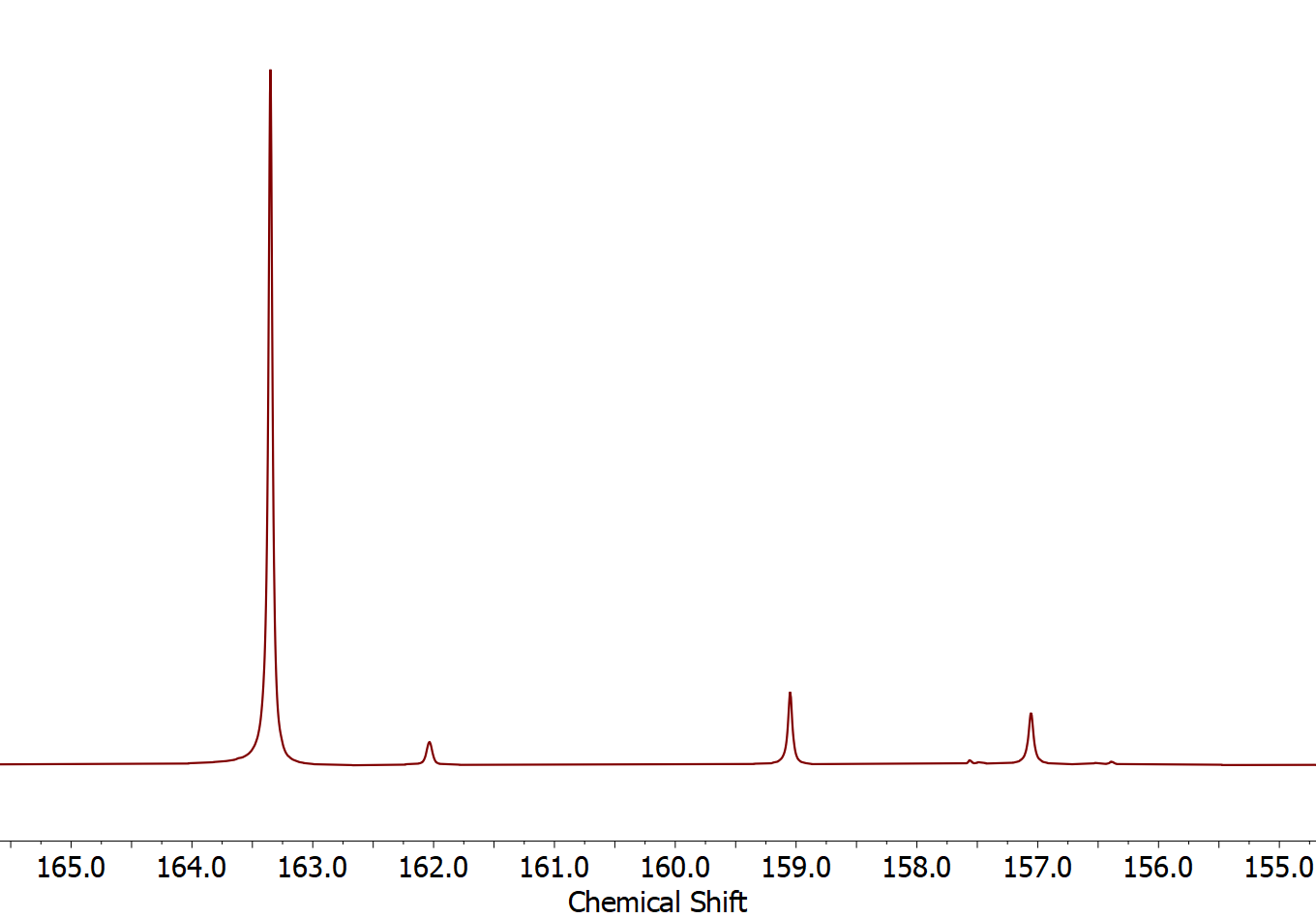


Figure S5. ^13^C NMR ^13^C labeled RuBpy-urea dissolved in CDCl_3_.


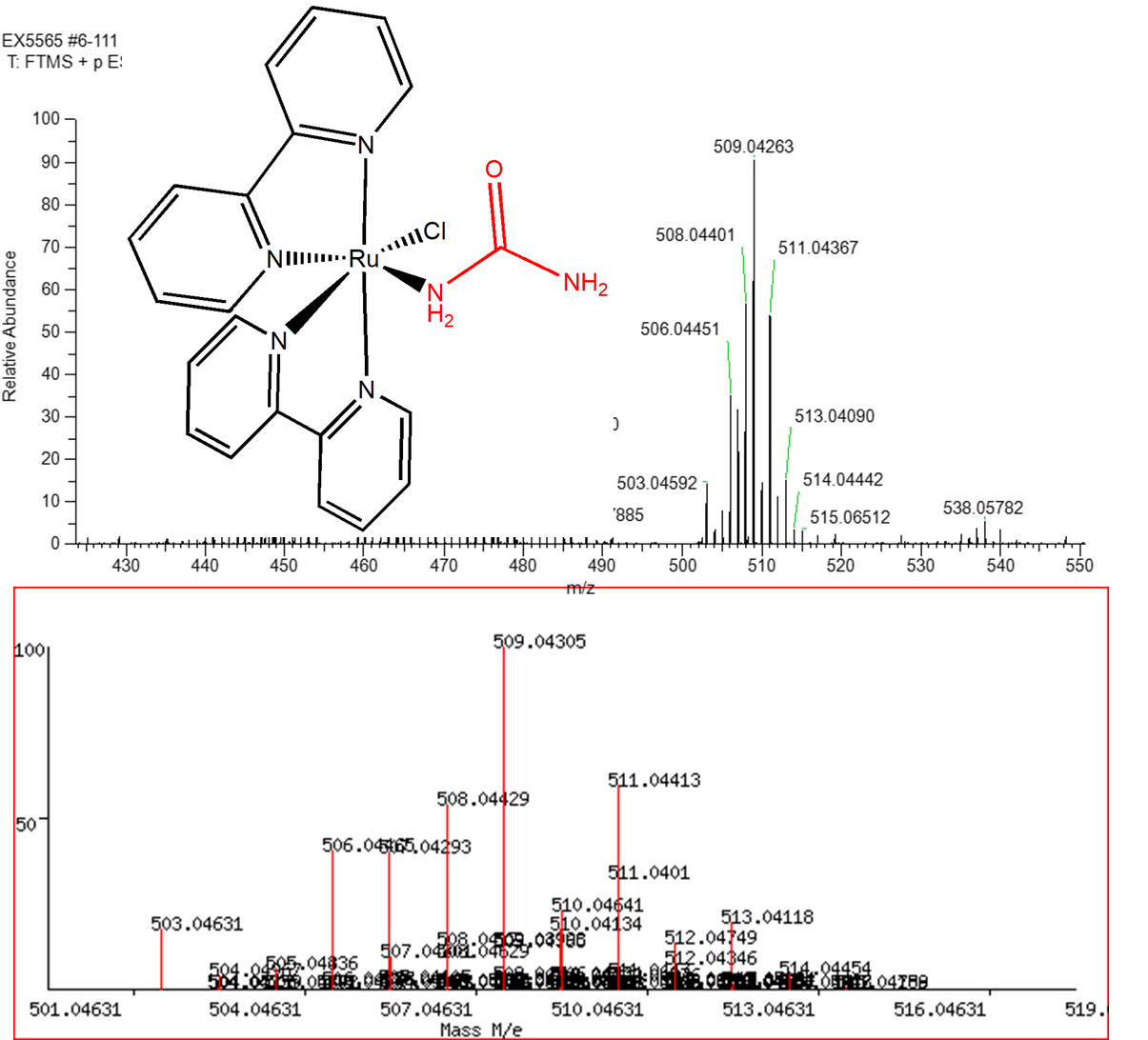


Figure S6. HRMS of RuBpy-urea. The complex was dissolved in acetone and diluted in methanol prior to injection. The positive mode HRMS evidences the presence of peaks with isotopic pattern centered at 509.04263 (left), which corresponds to a molecular formula of C_21_H_20_ClN_6_ORu, with theoretical isotopic pattern centered at a m/z peak of 509.04305 (right).


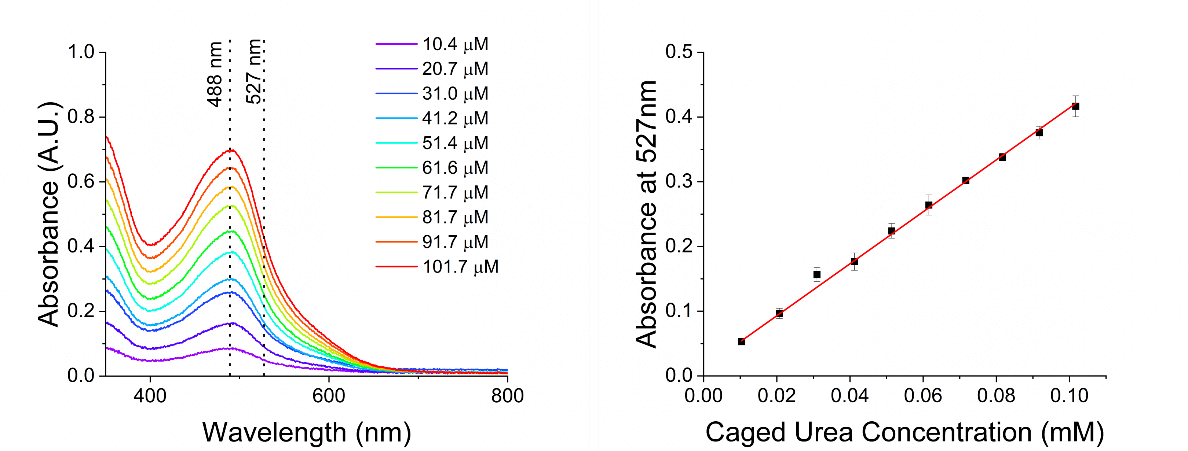


Figure S7. Electronic spectroscopy in the UV-Vis region of solutions of RuBpy-urea in 50 mM Tris-HCl pH 7.5. These solutions were obtained from the addition of different volumes of a 4.6 mM solution of RuBpy-urea in acetone to a Tris-HCl solution.

Figure S8. The B3LYP-D3(BJ)/def2-TZVP-CPCM(water) optimized structure of the: (A) *cis*- RuBpy-urea and (B) *trans*- RuBpy-urea. Carbon atoms are shown in gray, nitrogen in blue, oxygen in red, hydrogen in white, ruthenium in dark green, and chlorine in light green.


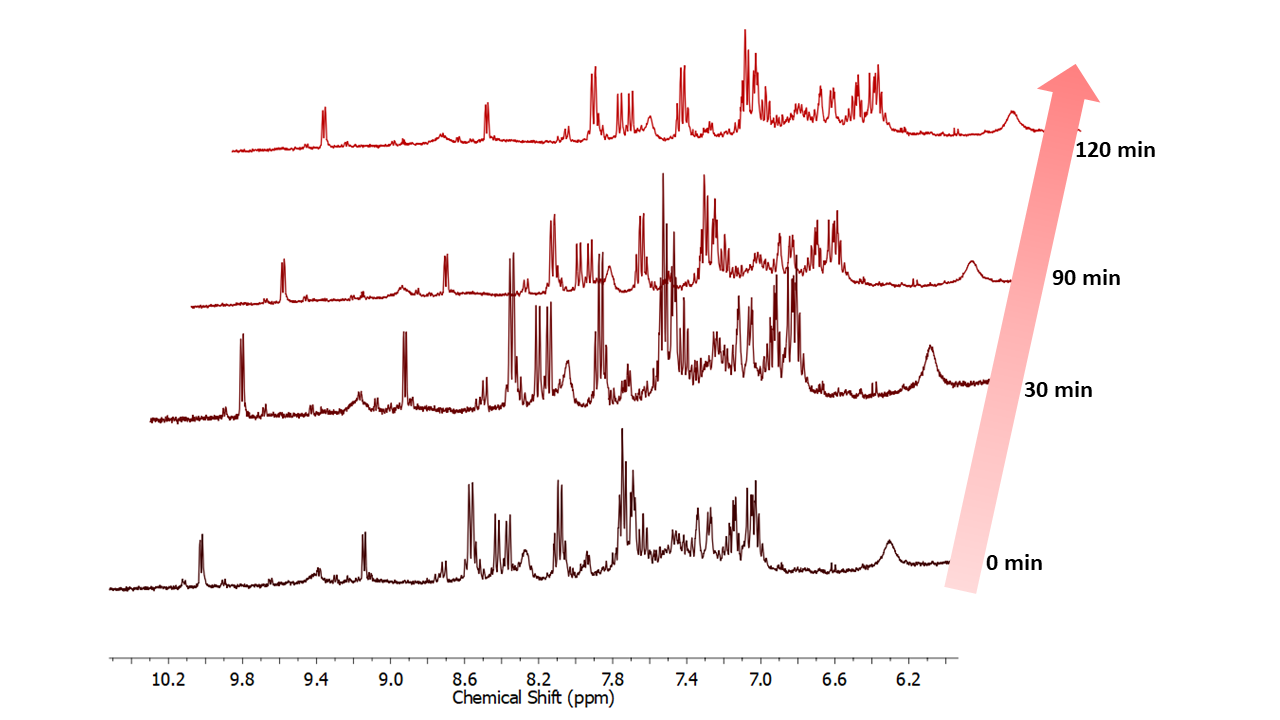


Figure S9. ^1^H NMR stability test of RuBpy-urea in a 50% acetone, 50% D_2_O medium. The NMR tube was left in the dark during this analysis.


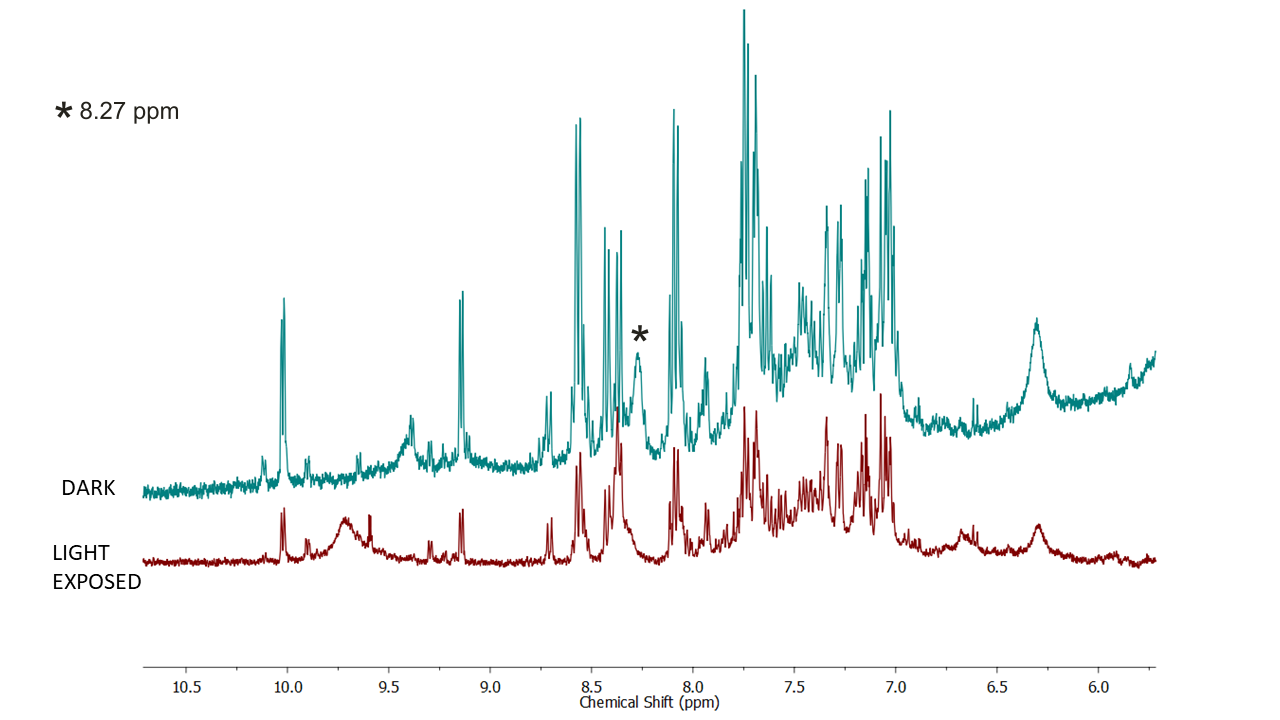


Figure S10. 1H NMR of dark and light exposed RuBpy-urea solution in a 50/50 solution of acetone and D_2_O.


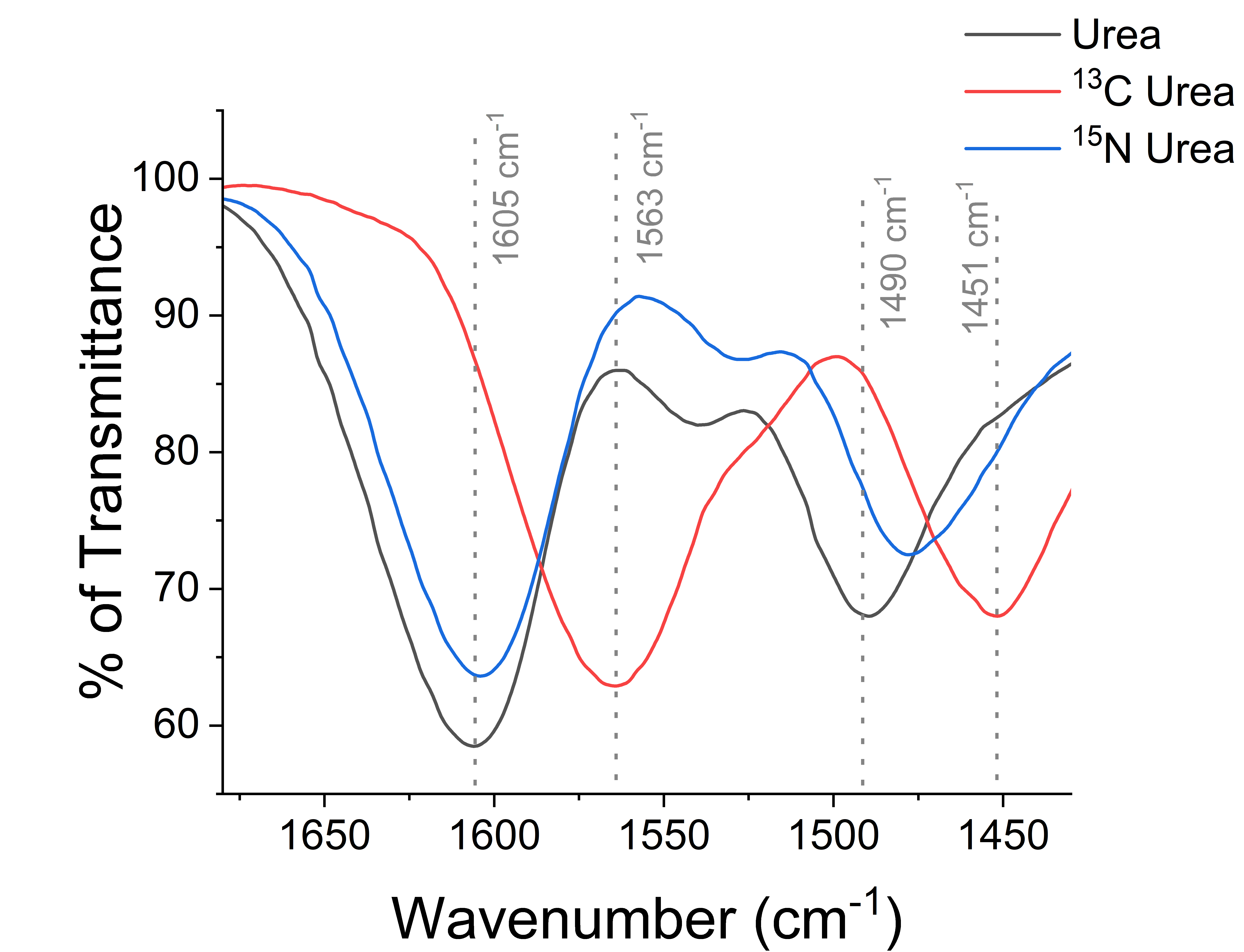


Figure S11. ATR-FTIR of the 1680-1420cm^-1^ region of urea (black line), ^13^C labeled urea (red line) and ^15^N labeled urea (blue line). All spectra were obtained from 10μL of 1M solutions in 75:25 (v/v) Tris-HCl (50mM pH 7.5) and acetone.


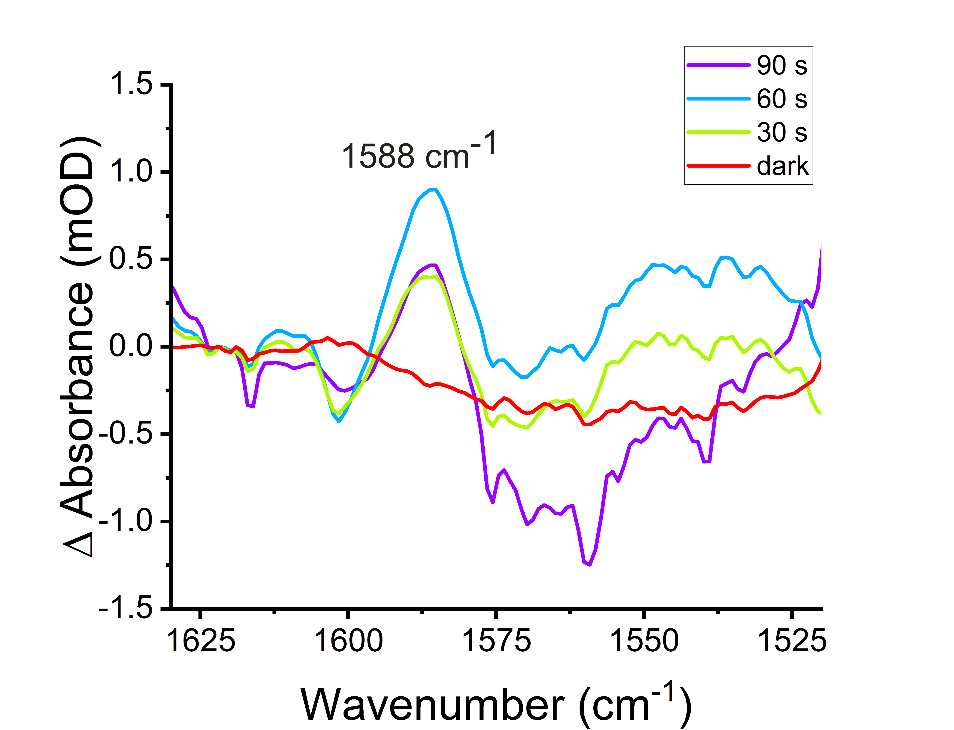


**Figure S12.** Phototitration of ^12^C RuBpy-urea monitored by FTIR at the region between 1630-1520cm^-1^. The experiments are obtained from photolysis of a thin film of the RuBpy-urea solution (in 75:25 (v/v) Tris-DCl (50 mM pD 7.3) and acetone) in a FTIR cell composed of CaCl_2_ windows and 50 μm Teflon spacer. Photolysis was performed with a crystal laser λ=527 nm (40.5 mW).

Figure S13. B3LYP-D3(BJ)/def2-TZVP-CPCM(water) infrared spectra of the urea molecule, RuBpy-urea and the related aquo complex.

Figure S14. Photolysis of RuBpy-urea at 70K. (A) FTIR in the region of 1680cm^-1^-1560cm^-1^ upon irradiation for different times. (B) Absorbance at 1604cm^-1^ versus time of irradiation, evidencing saturation at 400 s. (C) UV-Vis spectra of the cell used for photolysis, before (black line) and after irradiation (red line). The experiments are obtained from photolysis of a thin film of the RuBpy-urea solution (in 75:25 (v/v) Tris-DCl (50mM pH 7.3) and acetone) in a FTIR cell composed of CaCl_2_ windows and 50μm Teflon spacer. Photolysis was performed with a crystal laser λ=527nm (40.5mW).

**
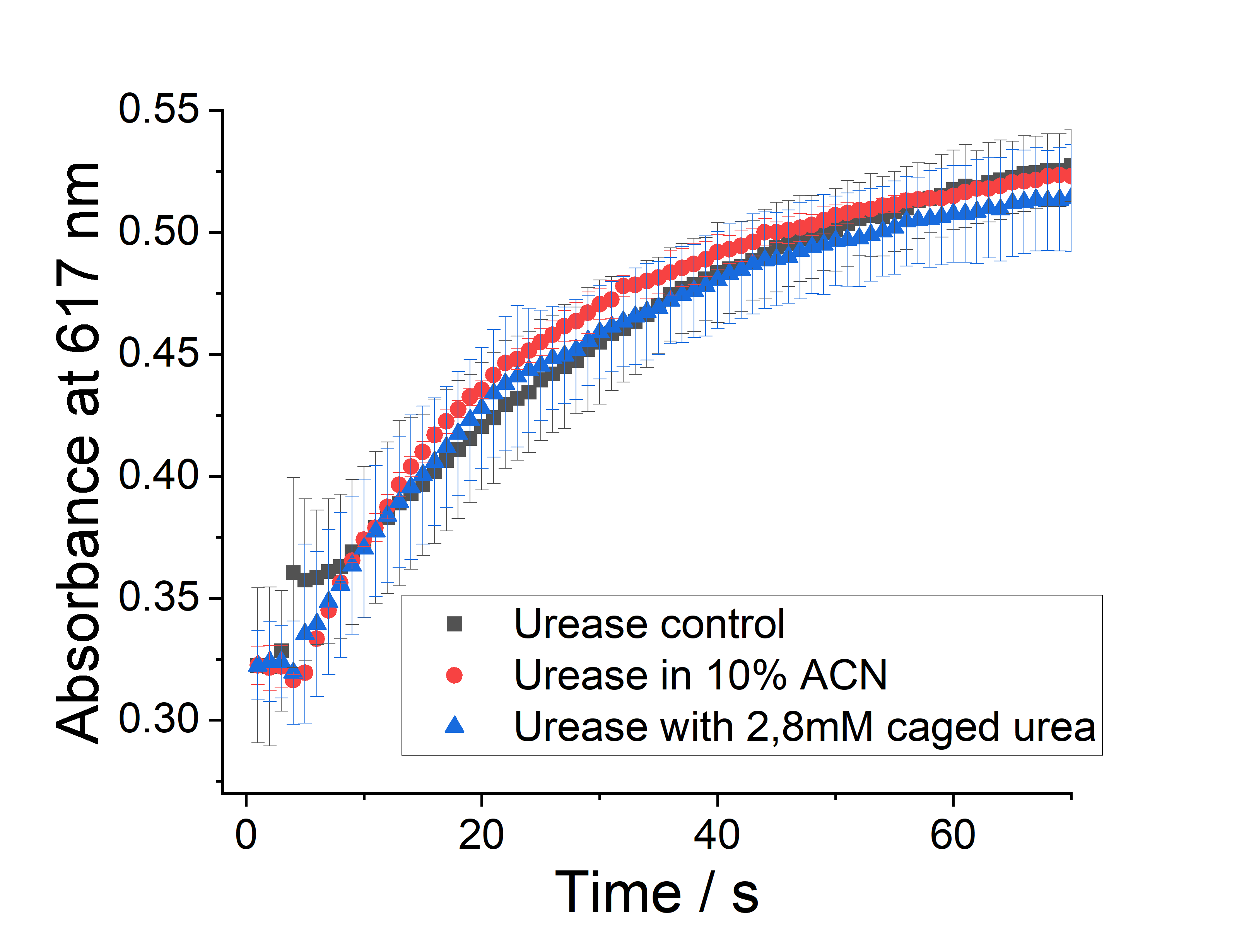
**

Figure S15. Urease kinetic assays using measured by the increase of the absorbance at 613 nm due to the increase of the reaction pH and the presence of the pH indicator bromo-thymol blue in the absence and presence of 10% acetonitrile and 2.8mM RuBpy-urea (dissolved in acetonitrile). The reaction was performed in 2.5mM HEPES pH 6.5 buffer, using 10 μM urease.


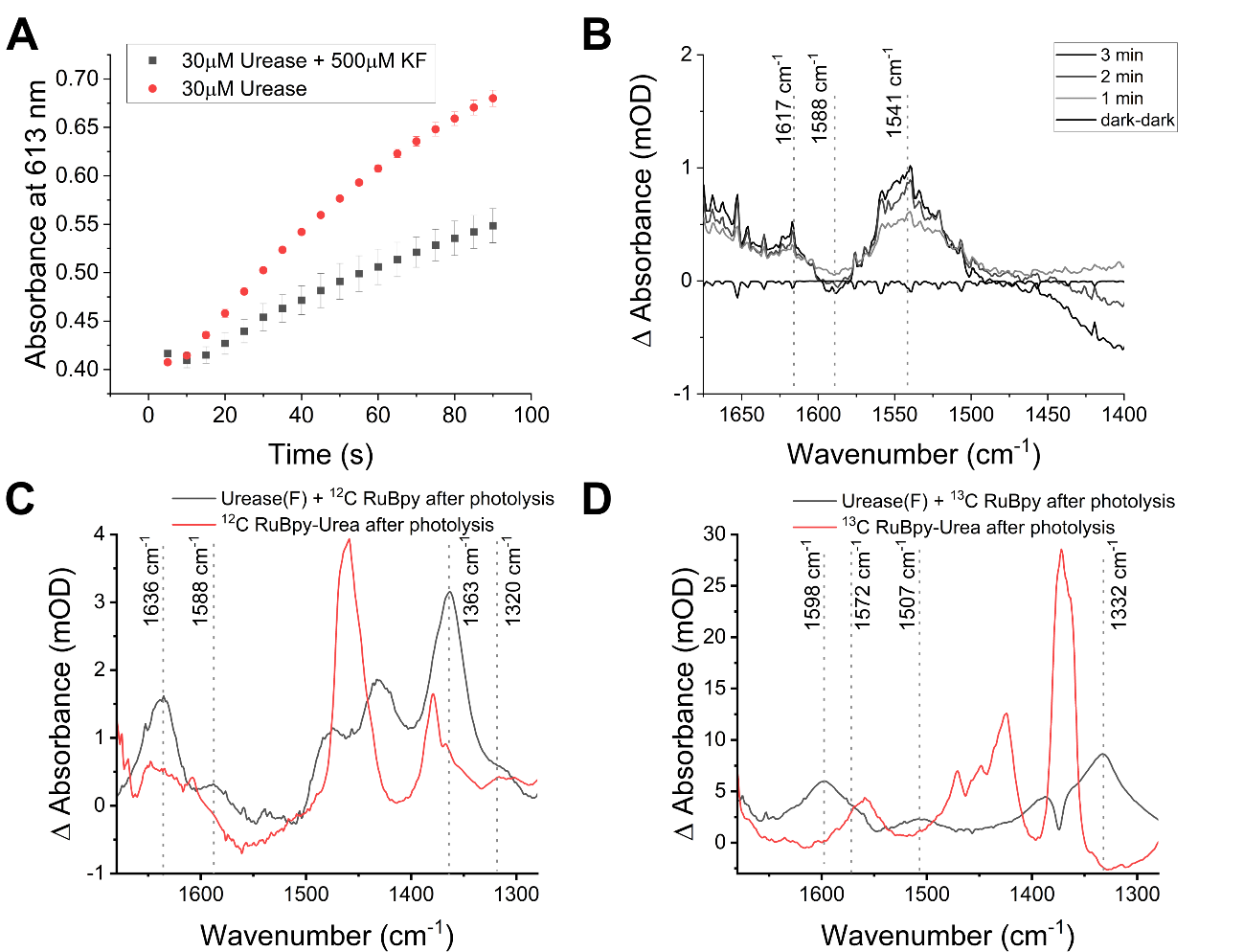


Figure S16. Experiments involving urease at room temperature. (A) urease kinetics measured by the increase of the absorbance at 613 nm due to the increase of the reaction pH and the presence of the pH indicator bromo-thymol blue, before and after addition of fluoride (2.5mM HEPES pH 6.5 buffer); (B) differential FTIR monitoring of urease(F) photolysis after 1 minute, 2 minutes and 3 minutes of irradiation; (C) Comparison of differential FTIR spectra (light-minus-dark) between photolyzed ^12^C-RuBpy-urea in the presence and absence of fluoride inhibited urease and (D) Comparison of differential FTIR spectra (light-minus-dark) between photolyzed ^13^C-RuBpy-urea in the presence and absence of fluoride inhibited urease. The photolysis experiments were obtained from photolysis of a thin film of the RuBpy-urea solution (10μL in acetone) and 0.8mM urease(F) (30μL in 0.1 M Tris-DCl pD 7.5) in a FTIR cell composed of CaCl_2_ windows and 50μm Teflon spacer. Photolysis was performed with a pulsed Q-switchted Nd:YLF laser (Crystal Laser) λ = 527nm (40 mW).


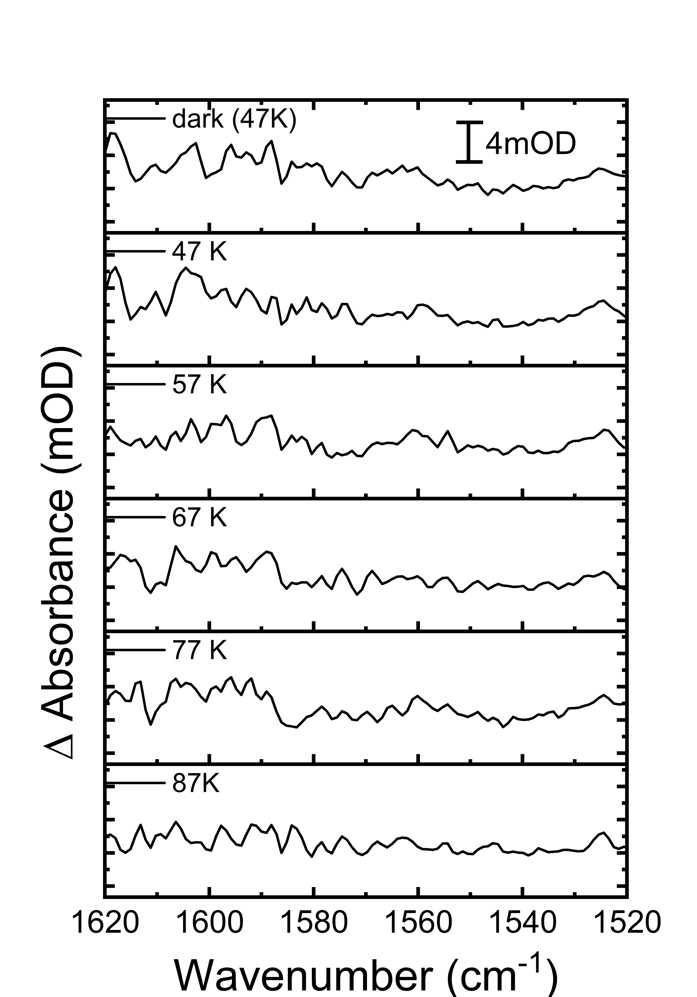


**Figure S17.** Cryo-FTIR of urease at pD 7.3 in the absence of RuBpy-urea. Differential FTIR under cryogenic conditions at different temperatures.


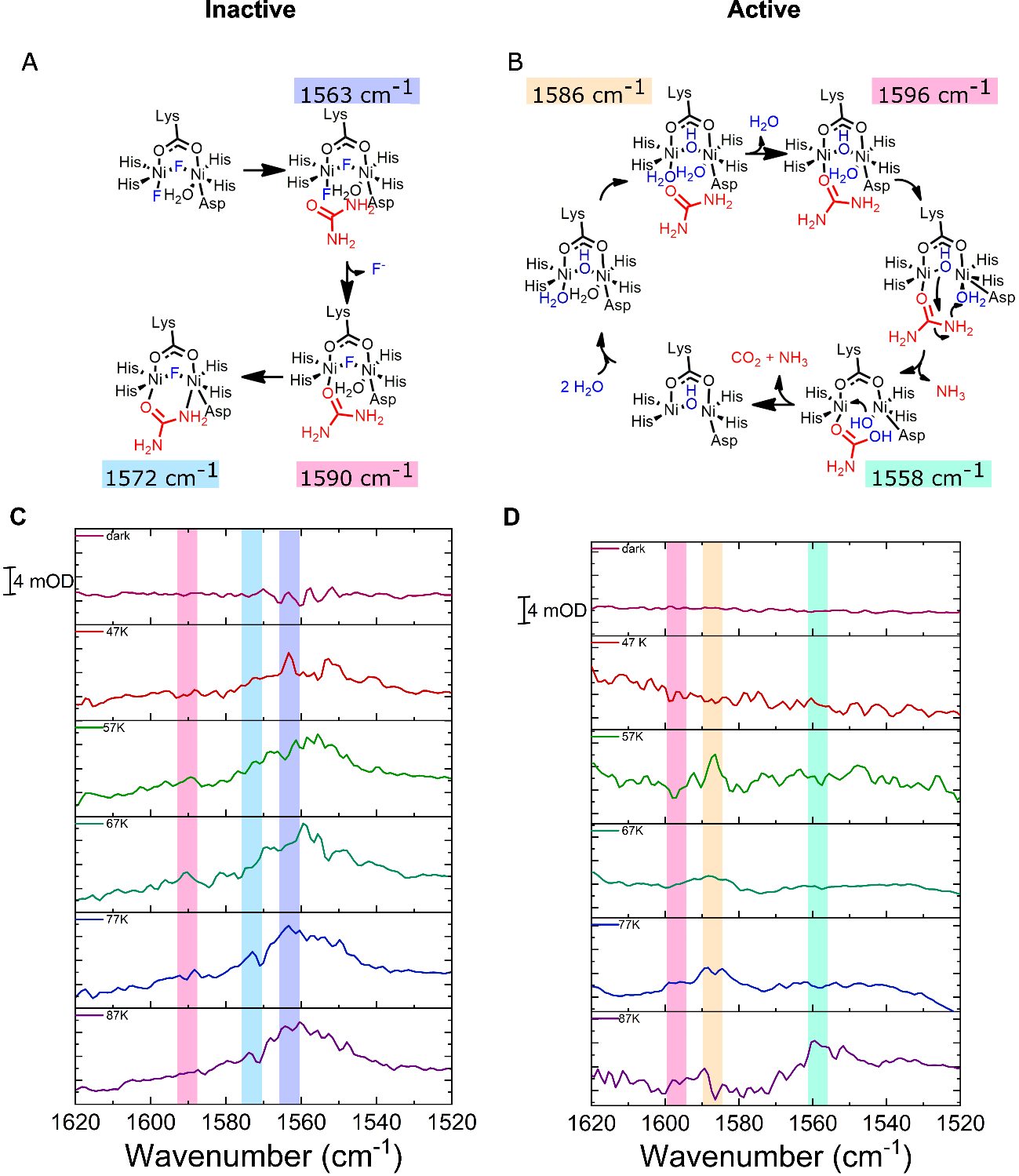


**Figure S18.** Cryo-FTIR experiments after RuBpy-urea photolysis. (A) Scheme of the reaction of the inactive urease with urea; (B) Scheme of the reaction of active urease with urea; (C) Differential FTIR under cryogenic conditions (light-minus-dark) at different temperatures for the active urease in the presence of ^13^C RuBpy-urea. The peaks are identified by colors: 1586 cm^-1^ (beige), 1596 cm^-1^ (pink) and 1558 cm^-1^ (blue), (D) Differential FTIR under cryogenic conditions (light-minus-dark) at different temperatures for active urease in the presence of ^13^C RuBpy-urea. The peaks are identified by colors: 1563 cm^-1^ (light purple), 1590 cm^-1^ (pink) and 1572 cm^-1^ (cyan) .


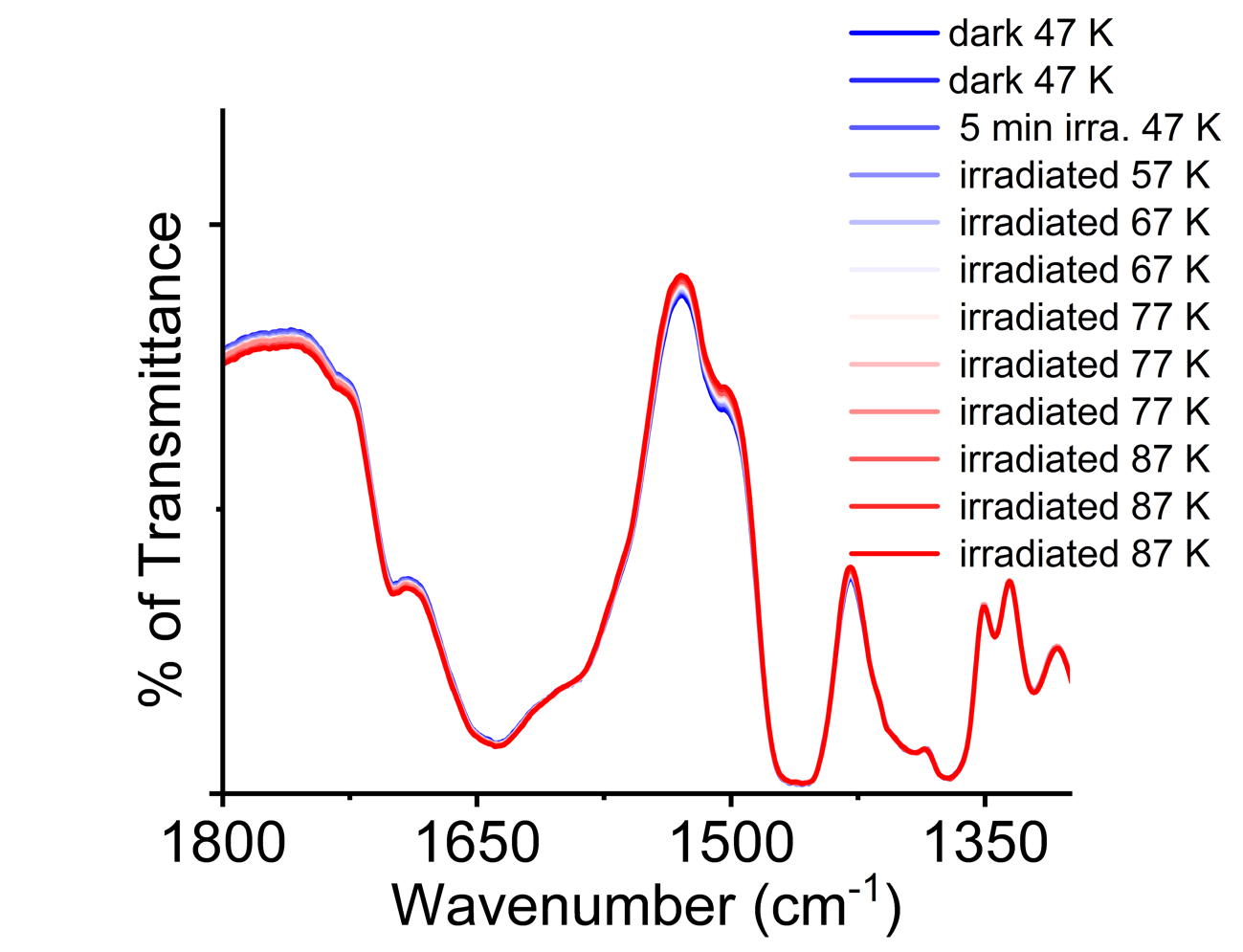


Figure S19. Broad range raw FT-IR spectra obtained at cryogenic temperatures. Evidencing changes observed between the initial spectrum and the irradiated spectra.


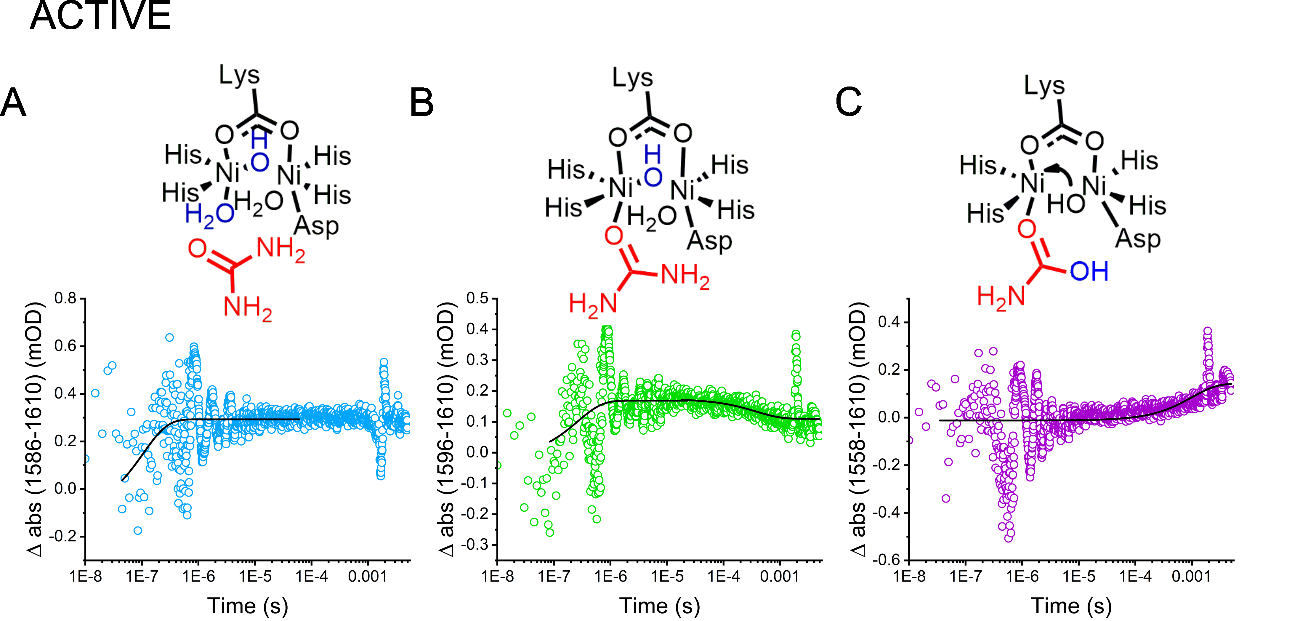


Figure S20. Transient infrared absorption experiment of the active urease in the presence of ^13^C RuBpy-urea. Each transient is corrected by subtraction of the solvent background obtained at 1610 cm^-1^. (A) absorption detected at 1586 cm^-1^ corresponding to the urea-urease encounter complex, (B) absorption detected at 1596 cm^-1^ corresponding to the κ:O coordination mode and (C) absorption detected at 1558 cm^-1^ corresponding to the coordinated carbamate.


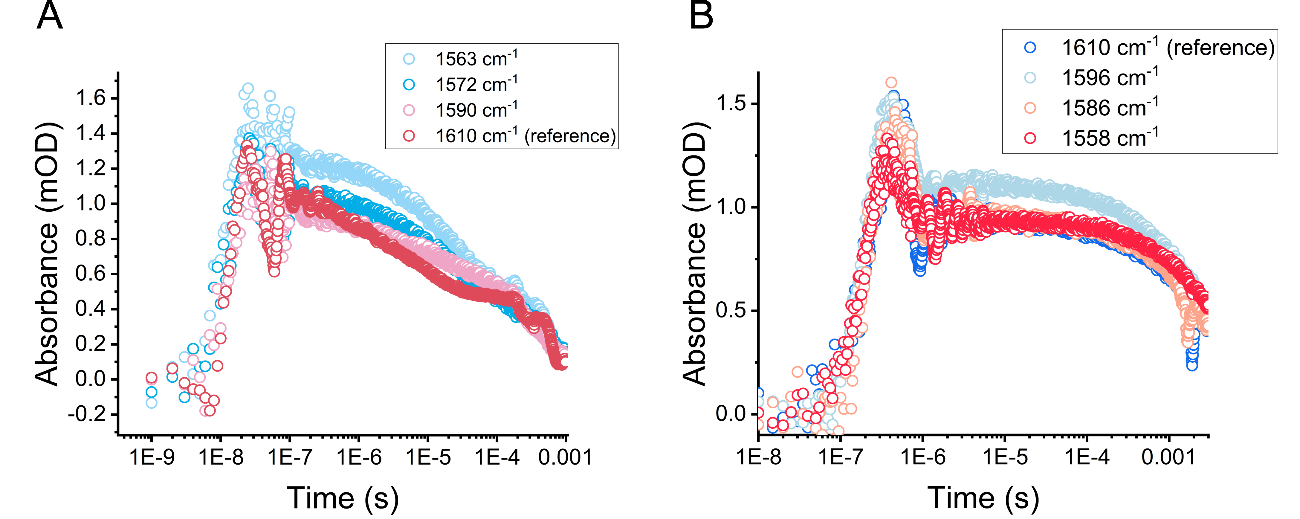


**Figure S21. Raw spectra obtained during the TRIR experiments**. (A) Transient absorbance of the inhibited urease in the presence of RuBpy-urea. The monitored absorbances were 1563, 1572, 1590 and 1610 cm-1, where 1610 cm^-1^ was used as a reference. (B) Transient absorbance of the inhibited urease in the presence of RuBpy-urea. The monitored absorbances were 1596, 1586, 1558 and 1610 cm-1, where 1610 cm^-1^ was used as a reference. All measurements were performed at room temperature.


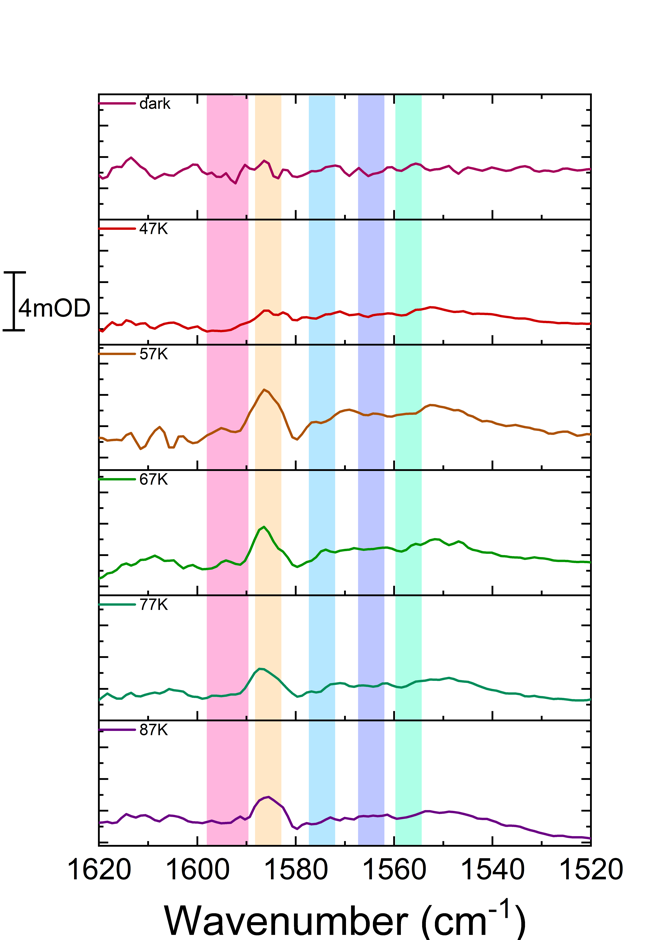


Figure S22. Cryo-FTIR of urease at pD 5.5 in the presence of ^13^C RuBpy-urea. Differential FTIR under cryogenic conditions at different temperatures. The peaks are identified by colors: 1563 cm^-1^ (blue), 1590 cm^-1^ (pink), 1572 cm^-1^ (cian),1586 cm^-1^ (beige), 1596 cm^-1^ (pink) and 1558 cm^-1^ (light green).


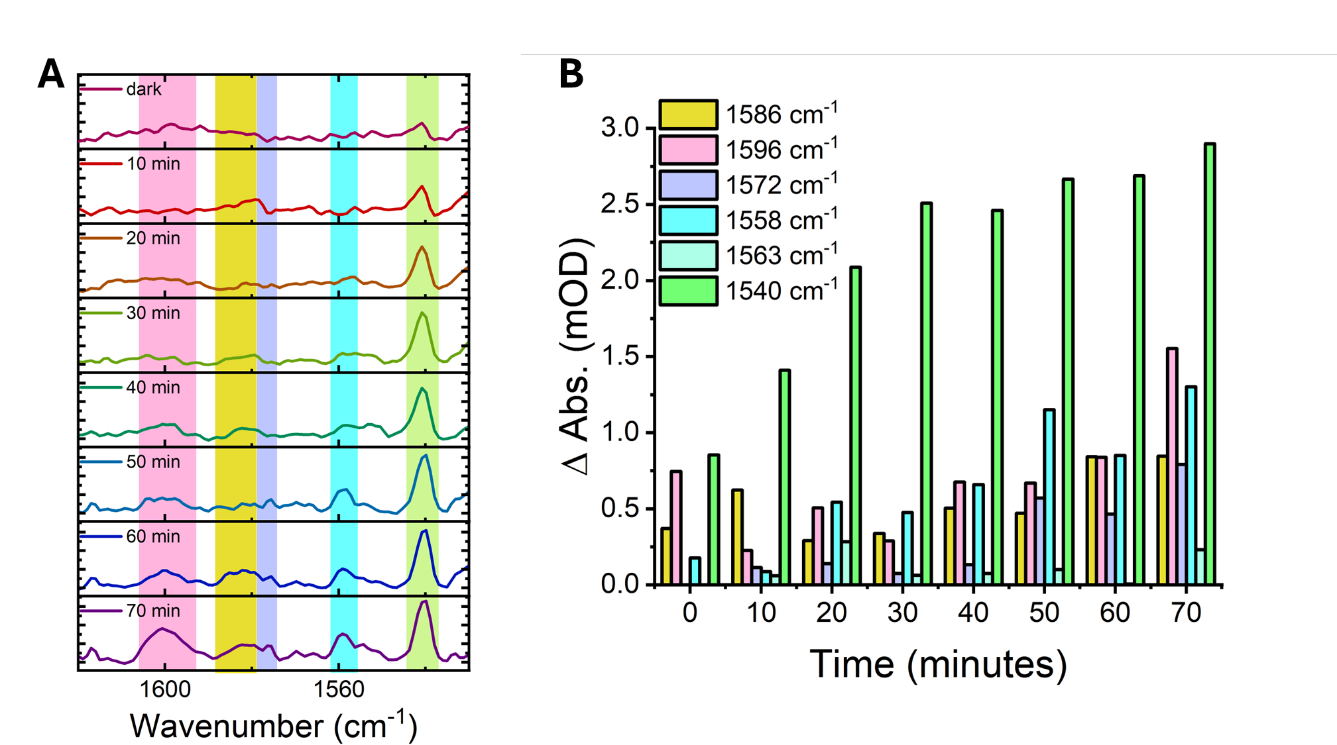


Figure S23. Cryo-FTIR of urease at pD 5.5 in the presence of ^13^C RuBpy-urea at constant temperature (77 K) and different reaction times. Differential FTIR under cryogenic conditions at different temperatures. The peaks are identified by colors: 1563 cm^-1^ (blue), 1596 cm^-1^ (pink), 1572 cm^-1^ (purple),1586 cm^-1^ (beige/yellow), and 1540 cm^-1^ (light green).

# 3. References

1. Zayat, L.; Noval, M. G.; Campi, J.; Calero, C. I.; Calvo, D. J.; Etchenique, R., A New Inorganic Photolabile Protecting Group for Highly Efficient Visible Light GABA Uncaging. *ChemBioChem* **2007,** *8* (17), 2035-2038.

2. Neese, F., Software update: the ORCA program system, version 4.0. *WIREs Comput. Mol. Sci.* **2017,** *8* (1), e1327.

3. Becke, A. D., Density-functional thermochemistry. III. The role of exact exchange  *J. Chem. Phys.* **1993,** *98* (7), 5648-5652.

4. Lee, C.; Yang, W.; Parr, R., Development of the Colle-Salvetti correlation-energy formula into a functional of the electron density. *Phys. Rev. B Condens. Matter.* **1988,** *37* (2), 785-789.

5. Vosko, S. H.; Wilk, L.; Nusair, M., Accurate spin-dependent electron liquid correlation energies for local spin density calculations: a critical analysis. *Can. J. Phys.* **1980,** *58* (8), 1200-1211.

6. Weigend, F., Accurate Coulomb-fitting basis sets for H to Rn†. *Phys. Chem. Chem. Phys.* **2006,** *8*, 1057-1065

7. Weigend, F.; Ahlrichs, R., Balanced basis sets of split valence, triple zeta valence and quadruple zeta valence quality for H to Rn: Design and assessment of accuracy. *Phys. Chem. Chem. Phys.* **2005,** *7*, 3297-3305

8. Aoto, Y. A.; Batista, A. P. d. L.; Köhn, A.; Oliveira-Filho, A. G. S. d., How To Arrive at Accurate Benchmark Values for Transition Metal Compounds: Computation or Experiment? *J. Chem. Theory Comput.* **2017,** *13* (11), 5291–5316.

9. Tang, S.-J.; Wang, M.-F.; Yang, R.; Liu, M.; Li, Q.-F.; Gao, F., More-Is-Better Strategy for Constructing Homoligand Polypyridyl Ruthenium Complexes as Photosensitizers for Infrared Two-Photon Photodynamic Therapy. *Inorg. Chem.* **2023,** *62* (21), 8210-8218.

10. Alp, M.; Yurdakul, S., Experimental and theoretical vibrational spectroscopic, quantum chemical analysis, and electronic properties investigations of novel ruthenium complexes (RuLCl2·2H2O; L: 4,4´-Dimethoxy-2,2´-Bipyridine, 4,4´-Dimethyl-2,2´-Bipyridine). *Polyhedron* **2023,** *234*, 116322.

11. Munshi, M. U.; Martens, J.; Berden, G.; Oomens, J., Vibrational Spectra of the Ruthenium–Tris-Bipyridine Dication and Its Reduced Form in Vacuo. *J. Phys. Chem. A* **2020,** *124* (12), 2449–2459.

12. Grimme, S.; Antony, J.; Ehrlich, S.; Krieg, H., A consistent and accurate ab initio parametrization of density functional dispersion correction (DFT-D) for the 94 elements H-Pu. *J. Chem. Phys.* **2010,** *132* (15), 154104.

13. Grimme, S.; Ehrlich, S.; Goerigk, L., Effect of the damping function in dispersion corrected density functional theory. *J. Comput. Chem.* **2011,** *32* (7), 1456-1465.

14. Barone, V.; Cossi, M., Quantum Calculation of Molecular Energies and Energy Gradients in Solution by a Conductor Solvent Model. *J. Phys. Chem. A* **1998,** *102* (11), 1995–2001.
